# The EGFR-ZNF263 signaling axis silences SIX3 in glioblastoma epigenetically

**DOI:** 10.1038/s41388-020-1206-7

**Published:** 2020-02-13

**Authors:** Zhibin Yu, Jianbo Feng, Wei Wang, Zhiyong Deng, Yan Zhang, Lan Xiao, Zeyou Wang, Changhong Liu, Qing Liu, Shuai Chen, Minghua Wu

**Affiliations:** 1 Hunan Provincial Tumor Hospital and the Affiliated Tumor Hospital of Xiangya Medical School, Central South University, Changsha, 410013 Hunan China; 2 Cancer Research Institute, School of Basic Medical Science, Central South University, Key Laboratory of Carcinogenesis and Cancer Invasion, Ministry of Education, Key Laboratory of Carcinogenesis, Ministry of Health, Changsha, 410078 Hunan China; 3 Yale Institute for Immune Metabolism, Shanghai Jiao Tong University School of Medicine, Shanghai, 200025 China; 4 Department of Pathology, Affiliated Hospital of Jining Medical University, Jining, 272000 Shandong China; 5 Second Xiangya Hospital, Central South University, Changsha, 410011 Hunan China; 6 Xiangya Hospital, Central South University, Changsha, 410008 Hunan China

**Keywords:** CNS cancer, Cancer genetics

## Abstract

The homeotic protein SIX3 is a transcription factor vital for neurogenesis and has a bivalent promoter. We previously showed that SIX3 can be transcriptionally silenced by DNA hypermethylation, functions as a tumor suppressor gene, and inhibits human glioblastoma transcriptionally. Here, we show that the activation of epidermal growth factor (EGFR) induces DNA methylation of SIX3 promoter through the MAPK pathway. ERK, when activated, binds with ZNF263, consequently abrogating the ubiquitination of ZNF263 and leading to its stabilization. ZNF263 binds to the core promoter region of SIX3 and recruits the KAP1/HATS/DNMT corepressor complex to induce transcriptional silencing of SIX3 through H3K27me3 and methylation of SIX3 promoter. Activation of the EGFR-ZNF263 signaling axis in phenotypically normal astrocytes or glioblastoma cells triggers or enhances tumorigenic activities, while elevated expression of the EGFR-ZNF263 signaling components in glioblastoma tissues is associated with poor prognosis of the patients. Together, our findings demonstrate that epigenetic silencing of SIX3 is controlled by a sophisticated and highly ordered oncogenic signaling pathway and therefore provide new insights into initiation and progression of glioblastoma.

## Introduction

During tumorigenesis, cells undergo a genome-wide epigenetic reprogramming process, which contributes to massive overall DNA hypomethylation and specific hypermethylation at certain CpG promoters [1, 2]. DNA methylation controls the transcription activity of the associated gene together with histone modifications [3–5]. Abundant tumor suppressor genes (TSG) are reportedly silenced by DNA methylation and histone modifications in human cancer [4, 6, 7]. However, little is known about the mechanisms for region-specific hypermethylation in cancer cells.

Glioblastoma is the most aggressive malignancy in central nervous system (CNS), with 15 months media survival [8]. Epigenetic alterations play critical roles in glioblastoma initiation and progression and give rise to various cells phenotypes [2, 9]. In high-grade pediatric gliomas, high frequency of H3.3K27M mutation led to the loss of suppressive H3K27me3 modifications [10]. DNA methylation alterations have been widely reported in gliomas [2, 11]. For instance, a CpG island methylator phenotype (G-CIMP) has been identified primarily in the secondary glioblastoma and leads to much improved survival of G-CIMP patients [12]. Mutations of isocitrate dehydrogenase 1 (IDH1) have been shown to be sufficient to establish G-CIMP by remodeling the methylome. While H3.3K27M, IDH1 mutations are detected in a small fraction of glioblastoma patients, the mechanism by which oncogenic mutations remodel the epigenome are still poorly understood [13–16].

It is well established that amplification and mutations of epidermal growth factor receptor (EGFR) are the most frequent genetic event in glioblastoma, which promotes tumor growth and survival through uncontrolled activation of signaling networks and metabolic reprogramming [15, 17–19]. Recent studies using integrated epigenome and transcriptome analyses revealed the critical role of EGFR in remodeling the epigenetic landscape of glioblastoma [15, 18]. EGFR hyperactivation has been found to transcriptionally suppress the expression of DNA demethylase-TET oncogene family member 1 (TET1), which contributes to the hypermethylation in the promoter region of a panel of TSGs in lung cancers and glioblastomas [20]. EGFR regulates histone modifications by promoting H3K23 acetylation, which enhances TRIM24 recruitment of STAT3 and activates downstream signaling [21]. Despite these findings, it is still unclear whether oncogenic EGFR initiates alterations of methylation of TSGs.

SIX3 belongs to the SIX transcription factor family and contains a homeobox domain for DNA binding and a six domain. SIX3 is predominantly expressed in CNS and critical for development [22–25]. SIX3 mutations correlate with multiple CNS developmental disorders, such as holoprosencephaly, aprosencephaly, and atelencephaly [26–28]. We have previously found that SIX3 is hypermethylated and acts as a suppressor in glioma by transcriptionally repressing AURKA/B [29]. SIX3 also inhibits the proliferation and invasion of glioblastoma cells by WNT pathway [23–25]. However, the mechanism underlying SIX3 hypermethylation in glioblastoma is not known. In this study, we revealed that epigenetic silencing of SIX3 is controlled by a highly ordered signaling pathway consisting of EGFR, ZNF263, and a subset of chromatin modifiers.

## Results

### SIX3 promoter hypermethylation correlates with low SIX3 expression in glioma

**Fig. 1:**
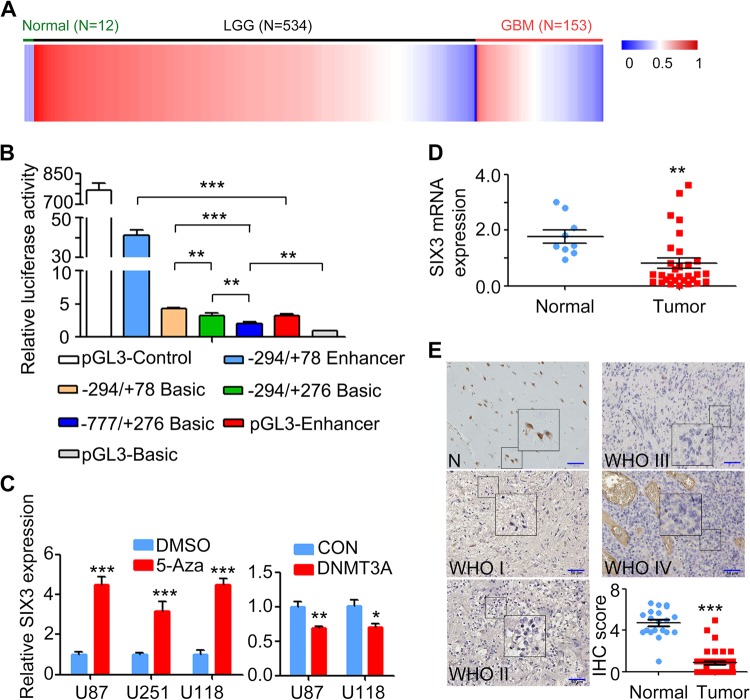
SIX3 hypermethylation correlates with low SIX3 expression in human glioma. **a** Experiments with TCGA showing DNA methylation levels in glioma tissues. **b** Luciferase reporter assay showing that −294/+78 (TSS = 0) is the core promoter regions of SIX3. **c** RT-qPCR analysis showing that 5-aza-2-deoxycytidine treatment enhances SIX3 expression (left), while overexpression of DNMT3A inhibits SIX3 expression (right). **d** RT-qPCR and (**e**) IHC analyses showing SIX3 expression in glioma and normal tissues. *p < 0.05; **p < 0.01; ***p < 0.001.

We had shown previously that SIX3 promoter contains higher levels of DNA 5-methylcytosine (5mC) in glioma than in normal brain tissues [29]. In this study, using the results from Gene Expression Omnibus (GEO) and The Cancer Genome Atlas (TCGA), we analyzed the level of methylation on the CpG island of SIX3 promoter and confirmed our earlier findings that SIX3 is hypermethylated in both low-grade glioma and glioblastoma (Fig. 1a, S1A). SIX3 is located at Chr2p21 and has an active promoter around the transcriptional start site (TSS) [30]. Luciferase reporter assay showed that the promoter region −294/+78 bp (TSS = 0) of SIX3 has the highest transcriptional activity (Fig. 1b). Using methylation-specific PCR (MSP) primers (Fig. S1B), we detected SIX3 DNA methylation in the phenotypically normal astrocyte cell line HEB and three glioblastoma cell lines (U251, U87, and U118), and the level of methylation correlated inversely with the expression of SIX3 (Fig. S1C, D). Treatment cells with DNA methyltransferase inhibitor 5-aza-2′-deoxycytidine reduced DNA methylation of SIX3 promoter and markedly enhanced its expression in glioblastoma cells (Fig. 1c). Overexpression of DNMT3A repressed SIX3 expression (Fig. 1c, S1E).

We next examined SIX3 expression in glioma from two independent sets of patients. qRT-PCR and IHC showed that SIX3 was expressed at a much lower level in tumor than in normal brain tissues (Fig. 1d, e) and that the expression was independent of World Health Organization histological grades (Fig. 1e). By analyzing datasets from TCGA, we found that SIX3 expression was lower in classic subtypes of glioblastoma than other subclasses or normal tissues (Fig. S1F). Intriguingly, it has been reported that the classic subtypes of glioblastoma are characterized by high frequencies of EGFR amplification and mutations [3].

### ZNF263 acts as a transcriptional repressor of SIX3

**Fig. 2:**
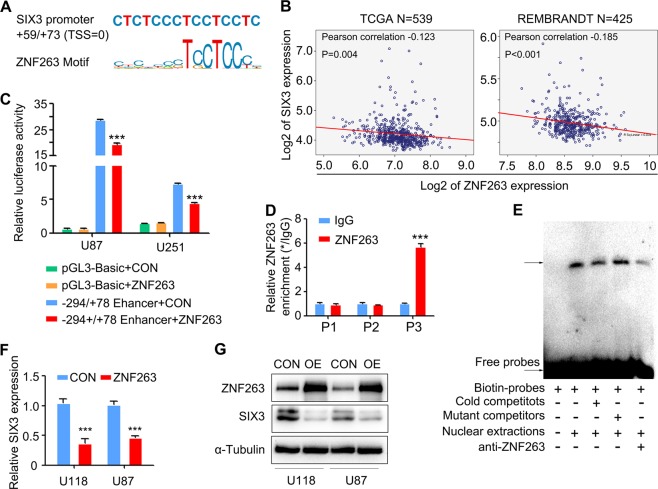
ZNF263 acts as a transcriptional repressor of SIX3. **a** Putative ZNF263 binding motif in SIX3 promoter. **b** Experiments with TCGA and REMBRANDT showing the inverse correlation between the ZNF263 and SIX3 levels in glioma tissues. **c** Luciferase reporter assay showing that ZNF263 suppresses the transcriptional activity of SIX3 promoter. **d** ChIP-qPCR analysis showing that ZNF263 binds to SIX3 promoter. **e** EMSA showing that ZNF3 binds to SIX3 promoter in vitro. **f** Immunoblotting and (**g**) RT-qPCR analyses showing that ZNF263 suppresses SIX3 expression. *p < 0.05; **p < 0.01; ***p < 0.001.

DNMT3A/B are the major de novo writers that directly catalyze the addition of methyl groups onto DNA and bind to DNA [31]. However, it remains undefined how DNMT3A/B target specific DNA sequences to initiate de novo DNA methylation. Recent studies showed that several transcription factors and lncRNAs regulate DNA methylation by binding to specific DNA sequences to recruit DNMTs and HMTs [1, 32, 33]. To identify the transcription factor(s) that might regulate DNA methylation of SIX3, we analyzed the sequences of SIX3 promoter and discovered a ZNF263 binding site in the core promoter region (Fig. 2a). ZNF263 belongs to the family of kruppel-associated box-containing zinc-finger protein (KRAB-ZNF) that contains the largest subset of the C2H2 zinc-finger proteins with 423 members [34, 35].

We first assessed the expression of ZNF263 in HEB cells and six primary patient-derived glioma cells and discovered an inverse relationship between ZNF263 and SIX3 (Fig. S2A). TCGA and REMRANDT datasets analysis also authenticated the result (Fig. 2b, S2B). We next examined potential binding of ZNF263 to SIX3 promoter. Co-transfection of ZNF263 expression vector with SIX3 −294/+78 luciferase reporter into U251 and U87 cells caused a significant decrease in SIX3 transcriptional activity compared with cells without ZNF263 expression (Fig. 2c). ChIP assay showed that ZNF263 bound to SIX3 promoter in region 3, not region 1 or region 2 (Fig. 2d). To further confirm the ZNF263 binding sequences, we synthesized biotin-labeled oligonucleotides containing ZNF263 binding motif within SIX3 promoter and allowed them to incubate with nuclear extracts. While the addition of wild-type competitors or ZNF263 antibody reduced the intensity of the shift in the protein–DNA complex, the addition of mutant oligonucleotide competitors failed to disrupt it (Fig. 2e). Finally, we examined the effects of ZNF263 depletion/overexpression on SIX3 transcription. Overexpression of ZNF263 in glioblastoma cells decreased the expression of SIX3 (Fig. 2f, g), while ZNF263 depletion markedly elevated SIX3 level (Fig. S2C–E). Thus, these results indicated that ZNF263 binds to SIX3 promoter and acts as a transcriptional repressor of SIX3.

### ZNF263 depletion decreases H3K27me3, H3K9me3, and DNA methylation of SIX3 promoter

**Fig. 3:**
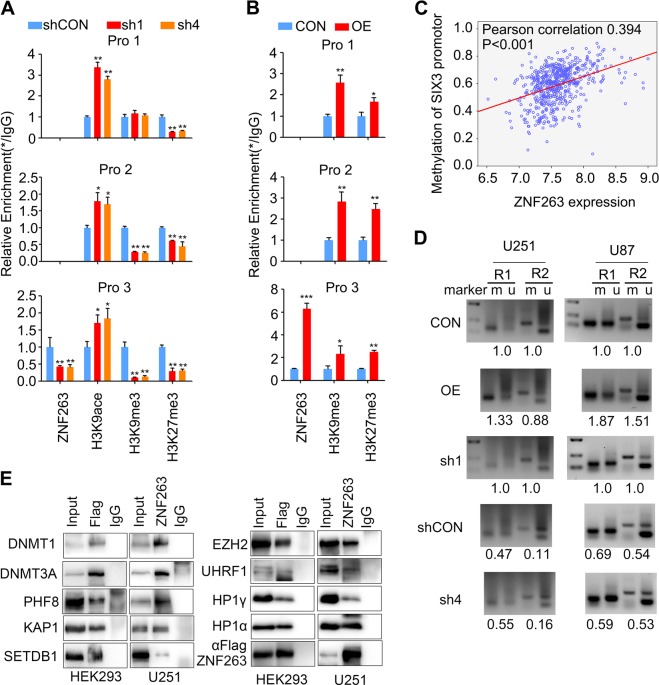
ZNF263 recruits chromatin modifiers to SIX3 promoter through protein–protein interactions. **a** ChIP-qPCR analysis showing that knockdown of ZNF263 enhances H3K9 acetylation and attenuates H3K9me3, H3K27me3 in SIX3 promoter. **b** ChIP-qPCR analysis showing that overexpression of ZNF263 promotes the enrichment of H3K9me3 and H3K27me3 in SIX3 promoter. **c** Experiments with TCGA showing that ZNF263 expression positively correlates with the DNA methylation level of SIX3 promotor. **d** MSP analysis showing that overexpression of ZNF263 elevates DNA methylation of SIX3 promoter, while knockdown of ZNF263 reduces DNA methylation level. Numbers represent the relative ratio of methylated DNA and unmethylated DNA. **e** IP analysis showing the interactions between ZNF263 and candidate proteins. HEK293 cells were transfected with ZNF63-FLAG and DNMT3A vectors. *p < 0.05; **p < 0.01; ***p < 0.001.

After revealing ZNF263 binding to and suppression of the transcriptional activity of SIX3 promoter, we next asked whether ZNF263 is involved in the regulation of DNA hypermethylation or histone modifications of SIX3 promoter. ChIP analysis showed that knockdown ZNF263 decreased the enrichment of H3K27me3 in the entire region of SIX3 promoter and specifically reduced the enrichment of H3K9me3 at region 2 and 3 (Fig. 3a). Overexpression of ZNF263 increased H3K27me3 recruitment to SIX3 promoter (Fig. 3b). Based on the well-established relationship between histone modifications and DNA methylation, we hypothesized that ZNF263 may affect DNA methylation of SIX3 promoter. By analyzing TCGA data, we found that ZNF263 expression correlated positively with SIX3 promoter methylation (Fig. 3c). Depletion or overexpression of ZNF263 decreased or increased the level of DNA methylation of SIX3, respectively (Fig. 3d).

It is well known that H3K27me3 and H3K9me3 are necessary for chromatin compaction and heterochromatin maintenance. The effects of ZNF263 on H3K27me3 and H3K9me3 led us to ask whether ZNF263 alter chromatin accessibility of SIX3 promoter in glioblastoma. We found that ectopic expression of ZNF263 significantly reduced the fraction of extractable SIX3 promoter, as shown by the formaldehyde-assisted isolation of regulatory elements (FAIRE) assay (Fig. S3A) while exerting little effect on GAPDH promoter. Conversely, a more extractable chromatin structure was observed upon knockdown ZNF263 (Fig. S3A). To test whether the effect of ZNF263 on chromatin compaction was specific, we assayed global chromatin compaction by probing chromatin accessibility using micrococcal nuclease (MNase) digestion. Knockdown or overexpression of ZNF263 did not cause notable alterations in the accessibility to MNase digestion, suggesting that ZNF263 does not affect global chromatin compaction (Fig. S3B).

### ZNF263 recruits chromatin modifiers to SIX3 promoter

How does ZNF263 regulate SIX3 epigenetically? We employed ZNF263 immunoprecipitation followed by tandem mass spectrometry (LC-MS/MS) to identify protein(s) interacting with ZNF263 (Fig. S4A). GO analysis showed that precipitated proteins are enriched in the cell nuclei and function in chromatin modifications, DNA transcription, and RNA splicing (Fig. S4B). Specifically, DNMT1, DNMT3A, and HDAC2 were present in the precipitates and support the role of ZNF263 in regulating DNA and histone modification of SIX3 promoter (Fig. S4C). In addition, TRIM28 (KAP1), UHRF1, PHF8, CBX3 (HP1α), CBX5 (HP1β), and MTA2, which have been shown to function in chromatin modifications, were also present as ZNF263-interacting proteins (Fig. S4C).

Immunoprecipitation followed by immunoblotting validated that ZNF263 interact with DNMT1, DNMT3A, HDAC2, KAP1, UHRF1, PHF8, HP1α, HP1β, and MTA2 (Fig. 3e) in U251 cells, which were highly consistent with those from LC-MS. Interestingly, although SETDB1 and EZH2 were not found via LC-MS, they were shown to interact with ZNF263 (Fig. 3e). No interactions were found between ZNF263 and HDAC1, SUZ12, SUV39H1, or SUV39H2 (Fig. S4D).

To elucidate ZNF263 function in recruiting chromatin modifiers to SIX3 promoter, we performed ChIP-qPCR analysis in U251 cells under overexpression or knockdown conditions of ZNF263. SUZ12, EZH2, DNMT3A, and RNA polymerase II antibodies were used for chromatin immunoprecipitation. Overexpression of ZNF263 led to increased enrichment of EZH2, DNMT3A, and SUZ12 to the promoter of SIX3, in accordance with decreased binding of RNA polymerase II (Fig. S4E). While knockdown of ZNF263 resulted in the opposite results, showing decreased assemblage of the SUZ12, EZH2, DNMT3A, and increased binding of Pol II at SIX3 promoter (Fig. S4F).

Finally, we asked whether ZNF263 affected the expression of DNMTs, H3K9 methyltransferase, and members of the PRC2 complex. The results from TCGA showed that ZNF263 expression had no significant correlation with the expression of DNMT3A, DNMT3B, SUV29H1, SUV39H2, EED, EZH2 at the mRNA level, while positively correlating with the expression of DNMT1, SETDB1, and SUZ12 (Fig. S4G). Moreover, immunoblotting showed that ZNF263 overexpression did not alter the levels of most proteins, with SETDB1 being the exception (Fig. S4H). Thus, ZNF263 mainly functions to direct the chromatin modifiers to the promoter region of SIX3 through protein–protein interactions without altering their expression level.

### The mitogen-activated protein kinase (MAPK) pathway is the main downstream effector of EGFR-mediated epigenetic silencing of SIX3

**Fig. 4:**
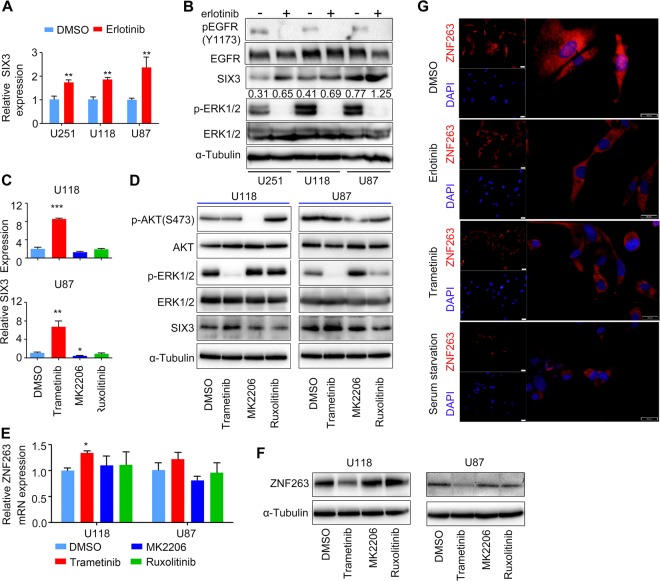
The MAPK pathway is the main downstream effector of EGFR-mediated epigenetic silencing of SIX3. **a** RT-qPCR and (**b**) immunoblotting analysis showing that erlotinib treatment rescues SIX3 expression. Numbers means relative SIX3 expression by densitometry. **c** RT-qPCR and (**d**) immunoblotting showing that inhibition of the MAPK/ERK pathway with Trametinib rescues SIX3 expression in astrocytoma cells, while treatment with MK2206 (a AKT inhibitor) and Ruxolitinib (a JAK inhibitor) fails to do so. **e** RT-qPCR analysis showing that Trametinib, MK2206, or Ruxolitinib had little effect on ZNF263 mRNA expression. **f** Immunoblotting showing that Trametinib treatment specifically downregulates ZNF263. **g** Immunofluorescence analysis showing that erlotinib or Trametinib reduces the accumulation of ZNF263 protein, especially in the nuclei. *p < 0.05; **p < 0.01; ***p < 0.001.

Earlier studies showed that EGFR and RAS induce epigenetic silencing of TSGs [20, 21, 34, 36]. Therefore, we asked whether SIX3 could be regulated by the EGFR pathway. We treated glioblastoma cells with erlotinib, a selective inhibitor of EGFR, which significantly increased SIX3 expression (Fig. 4a, b). We treated glioblastoma cells with EGF extrinsically and found that SIX3 was downregulated (Fig. S5A, B). It is well established that AKT, MAPK, and Janus kinase/signal transducer and activator of transcription (JAK/STAT) pathways can be activated upon EGFR activation. We therefore determined which pathway(s) may mediate epigenetic repression of SIX3 by using pharmacological inhibitors, namely Trametinib (a MEK inhibitor), MK2206 (an AKT inhibitor), or Ruxolitinib (a JAK inhibitor). Inhibition of AKT or JAK did not cause notable changes in SIX3 level (Fig. 4c, d, S5C). However, the inhibition of MAPK pathway markedly elevated SIX3 expression (Fig. 4c, d). Trametinib also significantly increased the transcriptional activity of SIX3 promoter (Fig. S5D). The results from mRNA half-life analysis showed that Trametinib did not affect the stability of the SIX3 mRNA (Fig. S5E).

Next, we further detected whether elevated SIX3 expression caused by Trametinib resulted in changes in epigenetic modifications of SIX3 promoter. In keeping with elevated SIX3 expression, Trametinib dramatically reduced the enrichment of H3K27me3 and H3K9me3 in SIX3 promoter and enhanced RNA pol II recruitment (Fig. S5F).In contrast, administration of extrinsic EGF strongly increased H3K27me3 and H3K9me3 modifications, and decreased RNA pol II recruitment (Fig. S5F). We also examined the DNA methylation change of SIX3 core promoter and discovered similar tendencies (Fig. S5G).

### MAPK inhibition promotes ZNF263 degradation through the ubiquitin pathway

Because ZNF263 plays a critical role in epigenetic silencing of SIX3 in glioblastoma, we next examined whether erlotinib or Trametinib induced SIX3 expression through the regulation of ZNF263. We found that erlortinib did not decrease and instead slightly elevated mRNA levels of ZNF263 in glioblastoma cells (Fig. S6A). Interestingly, the protein level of ZNF263 was significantly reduced upon EGFR inhibition (Fig. S6B). Conversely, the addition of EGF led to a substantial increase of ZNF263 protein levels (Fig. S6B). Similar to erlotinib, the MEK inhibitor Trametinib slightly increased ZNF263 mRNA levels (Fig. 4e), but markedly decreased ZNF263 protein levels (Fig. 4f). Inhibition of the AKT or JAK pathways exerted no effect on ZNF263 expression neither at the transcriptional or posttranscriptional level (Fig. 4e, f). Intriguingly, Trametinib appeared to specifically reduce ZNF263 protein levels without altering those of KAP1, SUV39H2, EED, or SETDB1, implying that the activation of EGFR/MAPK led to SIX3 silencing in glioblastoma mainly through the regulation of ZNF263 (Fig. S6C). Moreover, treatment of U251 cells with erlotinib, Trametinib led to the downregulation of ZNF263 protein, especially in the nuclei (Fig. 4g). Similar results were further confirmed in primary astrocytoma cells derived from patients (Fig. S6D, E). Together, these results suggested that the EGFR/MAPK cascade regulates ZNF263 at the protein level, not at the mRNA level.

**Fig. 5:**
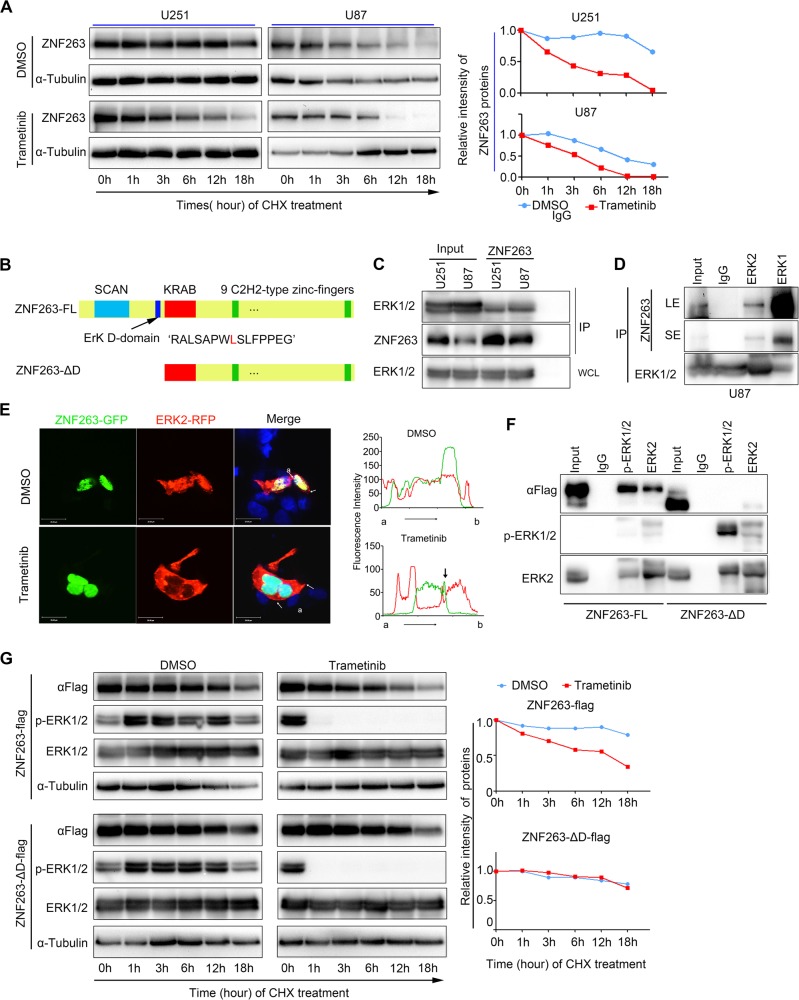
MAPK inhibition promotes ZNF263 degradation through the ubiquitin pathway. **a** Immunoblotting revealing the half-life of ZNF263 protein upon treatment with Trametinib. **b** The structure of full-length ZNF263 and ZNF263-ΔD (lacking the SCAN and D-domain). Experiments with IP followed by immunoblotting showing the interaction between ERK2 and ZNF263, either with ZNF263 antibody (**c**) or ERK2 antibody (**d**) for immunoprecipitation. **e** Representative confocal images of HEK293 cells co-transfected with ZNF263-GFP (green) and ERK2-RFP (red). The merged images show strong co-localization of ZNF263 and ERK2 in the nuclei (top). Trametinib treatment reduces the co-localization as indicated by the arrow (bottom). **f** IP and western blotting analysis showing p-ERK1/2 and ERK2 bind to full-length ZNF263 protein, but not ZNF263-ΔD. U251 cells were transfected with full-length ZNF263 and ZNF263-ΔD vectors, respectively. **g** Western blotting showing the half-life of full-length ZNF263 and ZNF263-ΔD protein with or without Trametinib treatment. *p < 0.05; **p < 0.01; ***p < 0.001.

How does the EGFR/MAPK pathway regulate ZNF263 protein? We first treated glioblastoma cells with cycloheximide to block de novo protein synthesis and then added Trametinib to inhibit the MAPK pathway, which resulted in a dramatic decrease in the half-life of ZNF263 (Fig. 5a). How ZNF263 protein degradation was mediated? We included inhibitors of autophagy including chloroquine, rapamycin, and everolimus and the proteasome inhibitor MG132 in the medium of glioblastoma cells with Trametinib treatment. MG132 treatment nearly completely rescued the decrease in ZNF263 protein levels, while the inhibition of autophagy failed to do so (Fig. S7A). We next asked whether modulation of the EGFR/MAPK pathway could alter the ubiquitination of ZNF263 and consequently its stability. HEK293 cells were transfected with expression vectors containing HA-tagged Ubiquitin (HA-Ubiquitin) and FLAG-tagged ZNF263 (ZNF263-FLAG) and were subsequently treated with Trametinib or EGF. Experiments with immunoprecipitation of ZNF263-FLAG followed by ubiquitin immunoblotting analysis revealed that inhibition of MAPK increased the fraction of ubiquitinated ZNF263, while activation of MAPK by EGF caused an opposite effect (Fig. S7B, C). We also showed that ubiquitination of ZNF263 was mediated by at lysine residue 63 (K63) (Fig. S7D). These results suggested that inhibiting EGF/MAPK pathway promotes ZNF263 degradation through the ubiquitin-proteasome pathway.

### Activated ERK2 interacts directly with ZNF263 through the ERK D-domain

By using Scansite (http://scansite.mit.edu), we discovered an ERK Docking domain (ERK D-domain) located between SCAN box and KRAB domain at N terminus of ZNF263 and predicted to bind to ERK1/2 (Fig. 5b). We conducted immunoprecipitation using antibody against ZNF263 in U251 and U118 cells followed by immunoblotting of ERK1/2, enabling us to find that ZNF263 co-immunoprecipitated with ERK2 (Fig. 5c). Reciprocally, ERK2, but not ERK1, was found to be co-immunoprecipitated with ZNF263 (Fig. 5d).

It is well known that ERK1/2, upon activation by MEK1/2 via phosphorylation, translocates to the nuclei [37]. We therefore asked whether phosphorylation of ERK is required for the interaction with ZNF263. We found that ZNF263 co-immunoprecipitated with p-ERK1/2 and ERK2 (Fig. S8A). We also observed high degree of co-localization between ZNF263 and ERK2 in the nuclei of U251 cells (Fig. 5e). Treatment of cells with Trametinib impaired ERK2 nuclear localization and also reduced ZNF263-GFP fluorescence in the nuclei (Fig. 5e).

Finally, we constructed a ZNF263 deletion mutant lacking the D domain (ZNF263-ΔD) and tagging it with either FLAG or GFP (Fig. S8B). We found that the D-domain deletion mutation did not alter ZNF263 translocation to the nuclei (Fig. S8C), but prevented its interaction with ERK2 (Fig. 5f). Next, we expressed FLAG-tagged ZNF263 and ZNF263-ΔD in U251 cells and subsequently treated the cells with cycloheximide. The ZNF263-ΔD mutant protein exhibited much better stability than the full-length ZNF263 when the cells were treated with the MEK1/2 inhibitor Trametinib (Fig. 5g). Thus, ERK1/2 interacts directly with ZNF263 through the ERK D-domain, and the interaction is vital for maintaining the stability of ZNF263 in glioblastoma cells.

### EGFR-mediated SIX3 silencing promotes glioblastoma development

It has been well documented that the gain of chromosome 7 where EGFR located is the first event in human glioblastoma development [38]. EGFR-vIII, a constitutively active deletion mutant of EGFR that constitutes a large portion of EGFR mutants, is a therapeutic target because of its strong role in enhancing tumorigenesis and malignant progression of glioblastoma [18]. To investigate the function of ZNF263 and SIX3 in the context of glioblastoma development, we first treated the phenotypically normal astrocytes (HEB cells) with high doses of EGF to mimic EGFR/MAPK hyperactivation during tumor initiation. HEB cells were treated with EGF for 10 days, and continuous activation of the EGFR/MAPK pathway by EGF markedly increased the level of ZNF263, while reducing that of SIX3 protein (Fig. S9A). In keeping with our earlier findings, EGF did not affect ZNF263 mRNA levels, but elevated ZNF263 protein levels in HEB cells (Fig. S9A, B). In contrast, SIX3 levels were reduced with increasing EGF concentrations at both the transcription and posttranscription level (Fig. S9A, B). Removal of EGF led to restored SIX3 expression and reduction of ZNF263 protein (Fig. S9C), while ectopic expression of EGFR-vIII in HEB cells led to elevated ZNF263 levels and reduced SIX3 levels (Fig. S9D). EGFR-vIII overexpression markedly increased anchorage-independent growth of HEB cells (Fig. S9E, F).

**Fig. 6:**
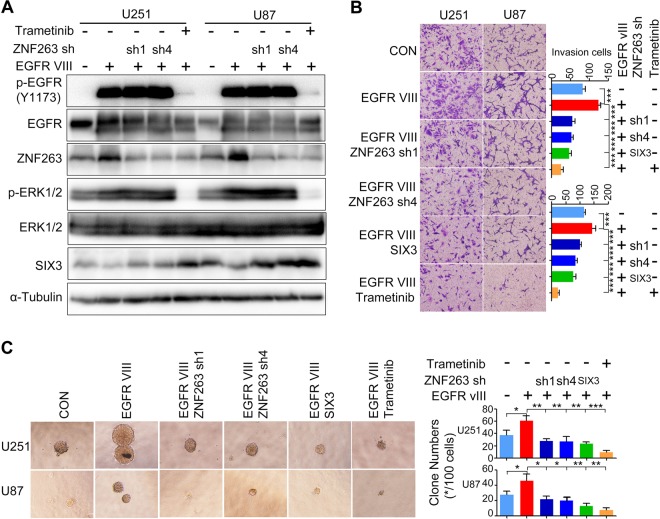
The function of the EGFR-ZNF263 signaling axis glioblastoma cells. **a** Immunoblotting showing that overexpression of EGFR-vIII increases ZNF263 expression, while decreasing SIX3 expression. Knockdown of ZNF263 or treatment with Trametinib restores SIX3 expression after overexpression of EGFR-vIII in human astrocytoma cells. **b** Transwell and (**c**) soft-agar colony formation assay showing that knockdown of ZNF263, overexpression of SIX3 or treatment with Trametinib decreases invasion and anchorage-independent growth of astrocytoma cells with EGFR-vIII overexpression. *p < 0.05; **p < 0.01; ***p < 0.001.

We also investigated the regulatory role of the EGFR-ZNF263 signaling axis in glioblastoma cells. We expressed EGFR-vIII in glioblastoma cells, which expectedly increased ZNF263 levels and decreased SIX3 levels (Fig. 6a). Knockdown of ZNF263 or Trametinib treatment rescued the downregulation of SIX3 (Fig. 6a). EGFR-vIII ectopic expression substantially enhanced invasion and anchorage-independent growth of glioblastoma cells (Fig. 6b, c). In contrast, ZNF263 knockdown, Trametinib treatment, or ectopic expression of SIX3 prevented the tumor-promoting effects of EGFR-vIII on glioblastoma cells (Fig. 6b, c). Together, these results indicated that SIX3 silencing induced by the EGFR-ZNF263 signaling axis has a functional role in glioblastoma malignant progression.

### Expression of the EGFR-ZNF263 signaling components in human glioblastoma tissues and the clinical outcomes

**Fig. 7:**
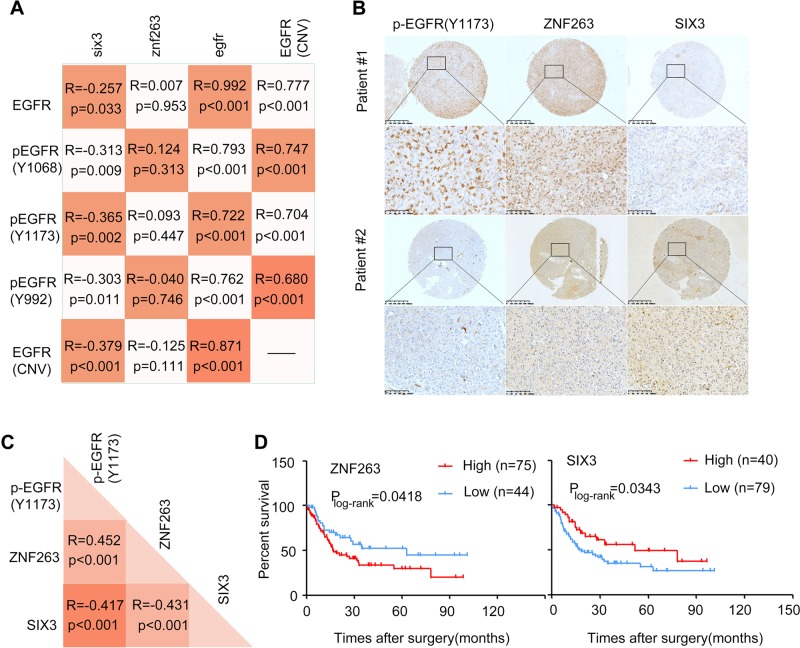
EGFR-ZNF263 signaling components in human glioblastoma tissues and the clinical outcomes. **a** Correlation analysis of EGFR, p-EGFR (Y1173), EGFR copy number variation (CNV), ZNF263 and SIX3 expression. Data from TCGA. **b** Representative images of IHC staining of p-EGFR (Y1173), ZNF263 and SIX3 from the glioma tissue microarray analysis. **c** The relationship between p-EGFR (Y1173), ZNF263 and SIX3 expression. **d** Kaplan–Meier survival curves comparing survival probability among ZNF263 high expression and low expression glioma patients (left), and SIX3 high expression and low expression glioma patients (right).

To further confirm the relationship of EGFR-ZNF263 signaling components, we investigated the reverse phase protein array data from TCGA which contains the expression data of EGFR, EGFR-pY1068, EGFR_pY1173, and EGFR_pY992 in part of glioblastoma samples. The association analysis of EGFR copy number, EGFR mRNA, EGFR protein, and phosphorylated EGFR showed high consistency by pairs (Fig. 7a). In addition, SIX3 expression has inverse correlation with EGFR copy number and protein expression, respectively. While no significant association between ZNF263 mRNA expression and EGFR copy number or protein expression has been found (Fig. 7a).

**Fig. 8:**
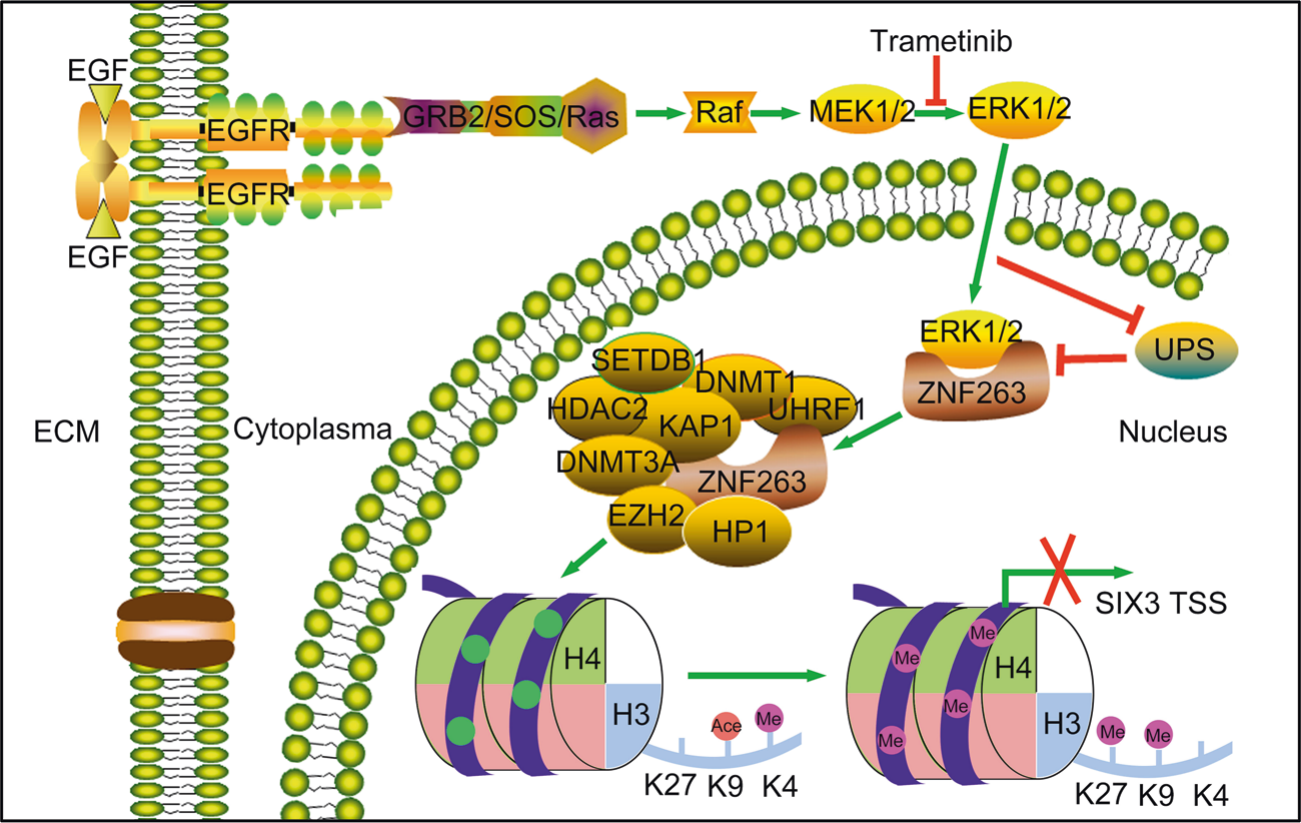
The EGFR-ZNF263 signaling axis silences SIX3 in glioblastoma epigenetically: a model.

We then extended our studies and determined the expression of EGFR-ZNF263 key signaling components in more glioma samples. We chose 235 glioma tissues and assembled them for tissue microarray analysis. One hundred sixteen patients were subjected to follow-up studies. We found strong positive correlation between the level of p-EGFR (Y1173) and that of ZNF263 (Fig. 7b, c). While SIX3 levels correlated inversely with the levels of both p-EGFR (Y1173) and ZNF263 (Fig. 7b, c). Survival analysis showed that the patients with high levels of p-EGFR exhibited much poorer prognosis than those with low p-EGFR levels (Fig. S10). Thus, both p-EGFR and ZNF263 can be used as an indicator for poor prognosis while SIX3 serves as an indicator for favorable prognosis of glioblastoma (Fig. 7d). Altogether, we conclude that epigenetic silencing of SIX3 is controlled by a sophisticated and highly ordered oncogenic signaling pathway and therefore provide new insights into initiation and progression of glioblastoma (Fig. 8).

## Discussion

DNA hypermethylation is a frequent mechanism for transcriptional silencing of TSGs in cancer [2, 10]. There are currently two schools of thoughts concerning the formation of tumor-specific hypermethylation. One argues that hypermethylation of TSGs results from a stochastic process, such as mutations of DNMTs or TETs, which causes extensive abnormalities in the methylome, while the cells with TSG hypermethylation are enabled with selectable growth advantages. The G-CIMP phenotype that is derived from IDH1/2 mutations caused by the metabolite 2HG-mediated suppression of TETs strongly supports this model [11, 20]. The other model suggests that hypermethylation of TSGs is regulated by specific signaling pathways consisting of a panel of components and in a highly ordered manner. Studies reported that activation of K-Ras and EGFR lead to region-specific hypermethylation of TGS and pro-apoptotic genes [1, 20, 32, 36, 39]. Michael R Green’ group recently showed that K-ras mutation leads to hypermethylation of Fas in NIH3T3 cells transformation, and contributes to epigenetic silencing of the Fas and INK4-ARF locus both through elaborated signaling pathways [1, 36]. In addition to the commonly known chromatin modifiers, a specific type of genes, the KRAB-ZNF factors are also involved in Ras/EGFR-mediated silencing of TSGs [1]. ZNF304 and ZNF354B are both involved in K-ras-mediated hypermethylation of INK4-ARF and Fas, respectively [36, 39]. Here, we firstly showed that EGFR/MAPK hyperactivation results in epigenetic silencing of SIX3 through ZNF263 in glioblastoma. We revealed a function for ZNF263 in transmitting oncogenic signals and subsequently targeting specific chromatin regions, followed by the recruitment of the corepressor complex KAP1/HATS/DNMT to the promoter regions of SIX3 and the resulting silencing of transcription. EGFR hyperactivation stabilizes ZNF263 protein through the MAPK pathway and subsequent suppression of ZNF263 ubiquitination. When ZNF263 proteins are in abundance, they can’t bind to the core promoter region of SIX3 and recruit DNMTs/HMTs to induce transcriptional silencing of SIX3 promoter. We also confirmed that ERK2, when activated/phosphorylated, directly binds to and phosphorylates ZNF263.

While we were analyzing the SIX3 promoter regions, it came to our attention that SIX3 contains a “bivalent” promoter. Bivalent chromatins are characterized by active histone modification H3K4me3 and suppressive H3K27me3 concurrently, leaving SIX3 poised for responding to extrinsic stimuli. The loss of H3K4me3 is more profound in mediating the silencing of bivalent chromatins in cancer than the gain of H3K27me3. Moreover, bivalent chromatins mark a high extent of promoters of genes encoding transcriptional factors known to regulate development [15, 18]. DNA hypermethylation is typically rarely found in these regions in ESCs and normal cells. However, in cancer cells, bivalent promoters are reported to be DNA methylated and silenced for a long term [20]. Our results found that SIX3 is hypermethylated in glioma, SIX3 promoter contains high levels of H3K27me3 in glioblastoma tissues, knockdown of SUZ12, a core subunit of PRC2 that catalyzes H3K27me3, fails to effectively rescue the level of SIX3 (Fig. S11), suggesting that DNA hypermethylation, but not H3K27me3, is responsible for epigenetic silencing of SIX3 in glioma.

Epigenetically based molecular classification of glioma uncovered that there exists G-CIMP (Glioma-CpG island methylator phenotype) subtype and NON G-CIMP subtype. G-CIMP is mainly found in low-grade gliomas and in subgroup of proneural subtype glioblastoma which was thought to be developed from low-grade glioma [12]. Strikingly, G-CIMP tumors possess high frequency of IDH1 mutation, which occurs in almost 80% G-CIMP glioblastoma and more than 70% low-grade gliomas [12, 14]. IDH1 mutant R132H (amino acid substitution at arginine 132 account for >95% IDH1 mutation in glioma), is further confirmed to induce the methylome which mirrors G-CIMP in human normal astrocytes [40]. The most popular IDH1 mutant R132H has been found to inactivate the enzyme’s ability to catalyze the conversion of isocitrate to α-KG (a-ketoglutarate) and gain the activity to catalyze α-KG into 2HG (2-hydroxyglutarate). Oncometabolite 2HG has been confirmed to competitively inhibit multiple α-KG-dependent dioxygenases, including histone demethylases, AlkB family, and TET family of 5mC hydroxylases [41]. IDH1 mutation in glioma leads to the loss α-KG and accumulation of 2HG, resulting in genome-wide histone and DNA methylation alterations. So, it is safe to suppose that IDH1 may affect SIX3 methylation and expression in glioma. When we investigated the data from TCGA, we found that IDH1 mutant LGG tumors harbor lower expression and higher-level promoter methylation of SIX3, in comparison with IDH1 wild-type LGG samples (Fig. S12C, D). While there is no difference of SIX3 between IDH1 mutant or wild-type glioblastoma. We further investigated the data of human normal astrocytes introduced with IDH1 mutant R132H [40]. The data showed that SIX3 expression in IDH1 mutant astrocytes is 1.28-fold of SIX3 expression in control astrocytes. Three probes target different CpG sites of SIX3 had been found to be differently methylated between IDH1 mutant and wild-type astrocytes. One CpG site located 800 bp upstream TSS and another one located in intron were hypermethylated in IDH1 mutant astrocytes. While the CpG site located 80 bp upstream TSS was hypomethylated in IDH1 mutant samples, and this site overlaps with the region R1 we detected and the core promoter region of SIX3 (Figs. S1B and S12E). Above all, IDH1 mutation can affect SIX3 methylation and expression through its global effect on reshaping histone and DNA methylome, rather than in a specific pattern.

The role of transcription factors in mediating the initiation of DNA methylation and histone modifications has been established [1, 36, 39]. DNMTs or PRC2 cannot recognize specific DNA sequences. In breast cancer, ZNF217 has been found to be essential for DNA hypermethylation of p15ink4b and does so by recruiting the CoREST complex to the promoter region [42]. The traditional view of DNA methylation-mediated transcriptional silencing is such that methylcytosine on the binding motifs of transcription factors directly blocks transcription factor binding to the chromatin, or indirectly prevents transcription factor binding due to their high-affinity binding for methyl-CpG binding-domain (MBD)-containing proteins [11]. However, recent studies showed that some transcription factors lacking MBD can still recognize and bind to methylated DNA. For example, ZFP57, a member of KRAB-ZNF family, recognizes methylated motif and is necessary for maintaining DNA methylation at the imprinting control regions in ESCs [43]. In this study, we found that ZNF263 interacts with DNMT3A, which is the main DNA methyltransferase for de novo DNA methylation, and with DNMT1, which is responsible for the maintenance of DNA methylation. Moreover, ZNF263 reportedly interacts with UHRF1, while UHRF1 interaction with DNMT1 remains to be the most profound mechanism for DNA methylation maintenance during replication. These findings suggest that ZNF263 not only recognizes unmethylated DNA and initiates the de novo methylation process, but also binds to the methylated motif and maintains DNA methylation. A better delineation of the function of ZNF263 in regulating chromatin modifications awaits future experimentation.

Since KRAB-ZNF proteins have been validated as indispensable factors linking signaling pathway with promoter methylation of TSGs, revealing the modulation of these factors is of great importance [36, 39, 40]. In this manuscript, we found that the stability of ZNF263 protein is maintained through EGFR/MAPK pathway. A hydrophobic motif ERK-Domain has been identified on the N terminal of ZNF263, which mediates the interaction with ERK1/2. Depletion of the N terminal completely hampered degradation of ZNF263 upon Trametinib treatment, which indicated that interaction between ERK and ZNF263 are vital for promoting ZNF263 protein stability. MAPKs are well-studied protein kinases that phosphorylate serine/threonine sites of their protein substrates when activated by extracellular signals. Upon stimulation, ERK1/2 are phosphorylated by MEK1/2, and translocated to nucleus, where they bind with and phosphorylate the nuclear substrates, especially transcription factors. Several TFs, such as Elk-1, GATA-1, p53, and c-Myc have been confirmed to be substrates of MAPKs [44–47]. As the reports showed, phosphorylation of c-Myc and p53 by MAPKs stabilized these factors and abrogated the degradation via the ubiquitin-proteasome system [46–48]. Here, we reported another ERK-Domain containing transcription factor ZNF263 as a potential substrate of MAPKs. In the mass-spectrum database, ten phosphorylation sites have been found in ZNF263 protein, and four sites are located around ERK D domain. Moreover, our mass-spectrum data showed several protein phosphatases binding with ZNF263, including PP1 and PP2A, which dephosphorylated and promoted degradation of c-Myc [49, 50]. Using Phospho-(Ser/Thr) antibody, we noticed that inhibition of MAPKs decreased the phosphorylated ZNF263 level (Fig. S7E). In combination of all these analyses, we can conclude that ZNF263 as a potential substrate of MAPKs, and phosphorylation of ZNF263 by MAPKs stabilizes ZNF263 protein from being degraded via UPPs. First and foremost, the mechanism of MAPK-ZNF263 axis can improve combination therapy strategy for glioma patients in future.

## Materials and methods

### Cell lines and human samples

Primary astrocytoma tissues and matched clinical data were obtained from the Department of Neurosurgery, the Xiangya Hospital, Central South University. Human glioma tissue array from glioma patients, who received surgery in Jining Affiliated Hospital of Jining Medical University from January 2008 to May 2017, was a gift from WW. All samples collected were obtained with the informed consent of the patients, and all experiments using human tissues were approved by the Joint Ethics Committee of the Central South University Health Authority. Astrocytoma cell lines U251, U87, and U118 were purchased from cell banks of Chinese Academy of Sciences (Shanghai, China). All astrocytoma cell lines were subjected to short tandem repeat test. Human phenotypically normal astrocyte cell line HEB was obtained from Guangzhou Institutes of Biomedicine and Health, Chinese Academy of Sciences (Guangzhou, China). HEB, U251, and U118 were cultured in high glucose DMEM with 10% FBS. U87 cells were cultured in MEM with 10% FBS at 37 °C with 5% CO_2_.

### Reagents and antibodies

5-Azacytidine (cat.A2385) and Cycloheximide (cat.R750107) were purchased from Sigma-Aldrich. Trametinib (cat.M1759), Chloroquine (cat. M2510), Rapamycin (cat.M1768), RAD001 (cat.M1909), and MG132 (cat.M1902) were purchased from Abmole. Erlotinib HCL (cat. S1023), MK2206 2HCL (cat.S1078), and Ruxolotinib (cat.S1378) were purchased from Selleck. The primary antibodies and their applications are listed in Supplementary Table 1.

### Plasmid construction

ZNF263 cDNA clone and shRNAs were purchased from GemeChem (Accession: NM_005741). We generated full-length and truncated ZNF263-FLAG-tagged fusion proteins using different primers. ERK2-RFP plasmid was constructed as previously described. The promoter regions of SIX3 were cloned from gDNA from normal human tissues and inserted into pGL3-basic and pGL3-enhancer vectors with T4 ligase.

### Luciferase reporter assay

Luciferase reporter assay was performed as previously described. To determine the activity of the core promoter region of SIX3, U251 cells were transfected with the firefly luciferase reporter plasmids and pRL-SV40 plasmids (Renilla luciferase) (at the ratio of 5:1). For repression analysis, the firefly luciferase reporter plasmids were transfected together with ZNF263 and pRL-SV40, or treated with Trametinib. Luciferase activity was determined according to the manufacturer’s protocol (Promega, E1910). The ratio of firefly to renilla was used to analyze the relative luciferase activity.

### Soft-agar colony formation assay

The soft-agar colony formation assay was performed as previously described [51]. Twenty four hours after transfection with EGFR-vIII plasmid, cells were dissociated and counted, seeded into six-well plates at 1000 cells/per well, and cultured for 1 month. The cells were subsequently fixed with 4% paraformaldehyde and stained with H&E. Colony numbers were counted by the use of ImageJ software. For soft-agar colony formation assay, 2 ml 6% low-melt agar with medium was added to the six-well plates. After 6% agar was solidified, 3% agar-cell mixture was added with the concentration of cells adjusted to 1 × 10^3^ cells/ml. After the medium was totally solidified, the plates were placed in cell incubator at 37 °C and 5% CO_2_ for ~20 days. The colonies per well were counted under the microscope.

### Genome DNA extraction and bisulfite conversion

Genome DNA extraction was performed according to the manufacturer’s protocol (9765, Takara). For DNA bisulfite conversion, standard protocol (59104, Qiagen) was used. One microgram gDNA was subjected to bisulfite conversion by using the standard conversion program.

### MSP

MSP was performed using a reaction mixture including 2× Taq MIX (10 μl), gDNA (2 μl), primers (1 μl), and H2O (7 μl) (total volume 20 μl). The PCR program includes degenerate reaction (95 °C, 5 min), degenerate reaction (95 °C, 30 s), annealing (52.6 °C, 2 min), extension (72 °C, 1.5 min, 40 cycles); and extension (72 °C, 10 min).

### Electrophoretic mobility shift assay

Electrophoretic mobility shift assay was performed as previously described [52], with slight optimization. The reaction buffer contained 1 mM HEPES (pH = 7.5), 5 μM EDTA, 2.5 mM NaCl, 10 μM DTT, 0.1% glycerol, 0.05 μg/μl Poly Id/Ic, and 0.05 μg/μl BSA.

### Immunohistochemistry and immunofluorescence

Immunohistochemistry and immunofluorescence analyses were performed as previously described [29]. For immunohistochemistry scoring, two independent pathologists were included. The staining intensity scored as 0, 1, 2, or 3 corresponding to negative, weak, intermediate, and strong brown staining, respectively. According to the positive stain cell numbers, 0, 1, 2, or 3, and 4 correspond to positive cells <5%, 25%, 50%, 75%, or >75%. Then intensity score with positive cell numbers score were added up, and mean of two individual scores from two pathologists were calculated and showed.

### Immunoprecipitation and tandem mass spectrometry

Precooled GLB Buffer (1 ml/10^7^ cells) was added to the washed cells, and 500 μl GLB (with prior addition of protease and phosphatase inhibitors) was added to the 10 cm culture dish and then lysed on ice for 10 min. Afterwards, the mixture was transferred to a 1.5 ml centrifugal tube and kept on the ice for 20 min for further lysis. During the period, the mixtures were subjected to several strong oscillations for 15 s or to ultrasonic perturbations for 10 s. After centrifugation at 13,000 × *g* (4 °C, 15 min), the supernatant was transferred to a new centrifugal tube, and the BCA method was used to determine the protein concentrations. A fraction of supernatant was used as input and stored at −80 °C. The rest of the supernatant was divided into several tubes based on protein concentration. Two micrograms of antibodies were added to each tube and the tubes were rotated gently at 4 °C overnight. After centrifugation, the samples were incubated with protein A/G magnetic beads rinsed with GLB buffer. The antigen/antibody mixtures were gently vortexed for 6 h at 4 °C or 1 h at room temperature. After centrifugation, the tubes was placed on a magnetic rack, and after the magnetic beads were separated, the supernatant was removed. The IP wash buffer was used to rinse the beads and was changed every 10 min for four times. The GLB buffer and loading buffer were subsequently used to re-suspend the magnetic bead-antigen/antibody mixtures, which were heated for 5 min at 95 °C and were subjected to immunoblotting as detailed below.

### Immunoblotting and chromatin immunoprecipitation assays

Immunoblotting and chromatin immunoprecipitation assays were performed as previously described [29].

### FAIRE and MNase digestion

The FAIRE and MNase digestion were performed as previously described [53, 54]. Cells were treated with 1% formaldehyde for 5 min at room temperature to form DNA–protein cross links, and cross-linking reaction was stopped by the addition of glycine a final concentration of 125 mM. The cells were pelleted, washed three times in 4 °C PBS, and lysed on ice for 10 min in cell lysis buffer (10 mM Tris-HCl, 10 mM NaCl, 3 mM, MgCl_2,_ 0.5% NP-40 and protease inhibitors). Nuclei were pelleted and lysed on ice for 10 min in cell lysis buffer (10 mM EDTA, 50 mM NaCl, 1% SDS and protease inhibitors). Lysates were sonicated in a sonic Bioruptor and diluted with 50% v/v dilution buffer (12 mM EDTA, 17 mM Tris-HCl, 167 mM NaCl, 0.01% Triton X-100, 0.01% SDS). Cell debris was removed by microcentrifugation, and free DNA was extracted from the collected supernatant by phenol/chloroform extraction. Under these conditions, DNA that is not cross-linked with the protein remains in the aqueous phase, while DNA that cross-links with the protein remains in the phenolic phase.

### Statistical analysis

The statistical analyses in this study were performed by the use of SPSS 22.0 and GraphPad Prism 5 software. Student *t* test and one-way ANOVA were used for comparison of data between two or more groups, and Kaplan–Meier survival curve was plotted using log rank. All data were calculated using mean ± standard deviation (mean SD). The experiments were repeated at least three times. All tests were two-tailed; *p* < 0.05 was considered statistically significant.

## Supplementary information

supplement figure and legend

supplement_antibody information

supplementary information
