# Supplementary material for: The EGFR-ZNF263 signaling axis silences SIX3 in glioblastoma epigenetically
Source: Oncogene. 2020 Feb 13;39(15):3163–78. doi: 10.1038/s41388-020-1206-7 (PMC7142014; doi:10.1038/s41388-020-1206-7)
Supplement: Supplementary file 1 — supplement figure and legend [file 41388_2020_1206_MOESM1_ESM.docx]

# The EGFR-ZNF263 Signaling Axis Silences SIX3 in Glioblastoma Epigenetically

Zhibin Yu^1,2,6^, JianboFeng^1,2^, Wei Wang^3^, Zhiyong Deng^1^, Yan Zhang^1,2^, Lan Xiao^2^, Zeyou Wang^4^, Changhong Liu^1,2^, Qing Liu^5^, Shuai Chen^1^*, Minghua Wu^1,2^*

1. Hunan Provincial Tumor Hospital and the Affiliated Tumor Hospital of Xiangya

Medical School, Central South University, Changsha 410013, Hunan, China

2. Cancer Research Institute, School of Basic Medical Science, Central South

University; Key Laboratory of Carcinogenesis and Cancer Invasion, Ministry of

Education; Key Laboratory of Carcinogenesis, Ministry of Health. Changsha 410078,

Hunan, China

3. Department of Pathology, Affiliated Hospital of Jining Medical School, Jining, 272000, Shandong, China

4. Second Xiangya Hospital, Central South University, Changsha 410011, Hunan, China

5. Xiangya Hospital, Central South University, Changsha 410008, Hunan, China

6. Shanghai Jiao Tong University School of Medicine - Yale Institute for Immune Metabolism, Shanghai Jiao Tong University School of Medicine, Shanghai, 200025, China.

**Running Title: The EGFR-ZNF263 axis silences SIX3 epigenetically**Note: Zhibin Yu and Jianbo Feng contributed equally to this article.

* To whom correspondence should be addressed:

Minghua Wu, Cancer Research Institute, Central South University, Changsha, Hunan 410078, Tel: 86-731-82355401. Fax: 86-731-82355401. E-mail: wumh@csu.edu.cn

Shuai Chen, Hunan Provincial Tumor Hospital and the Affiliated Tumor Hospital of Xiangya Medical School, Central South University ,Changsha 410013, Hunan, China Tel: 86-731-82355401. Fax: 86-731-82355401. E-mail: chenshuai16@139.com

**Supplemental Figures and figure legends**

**
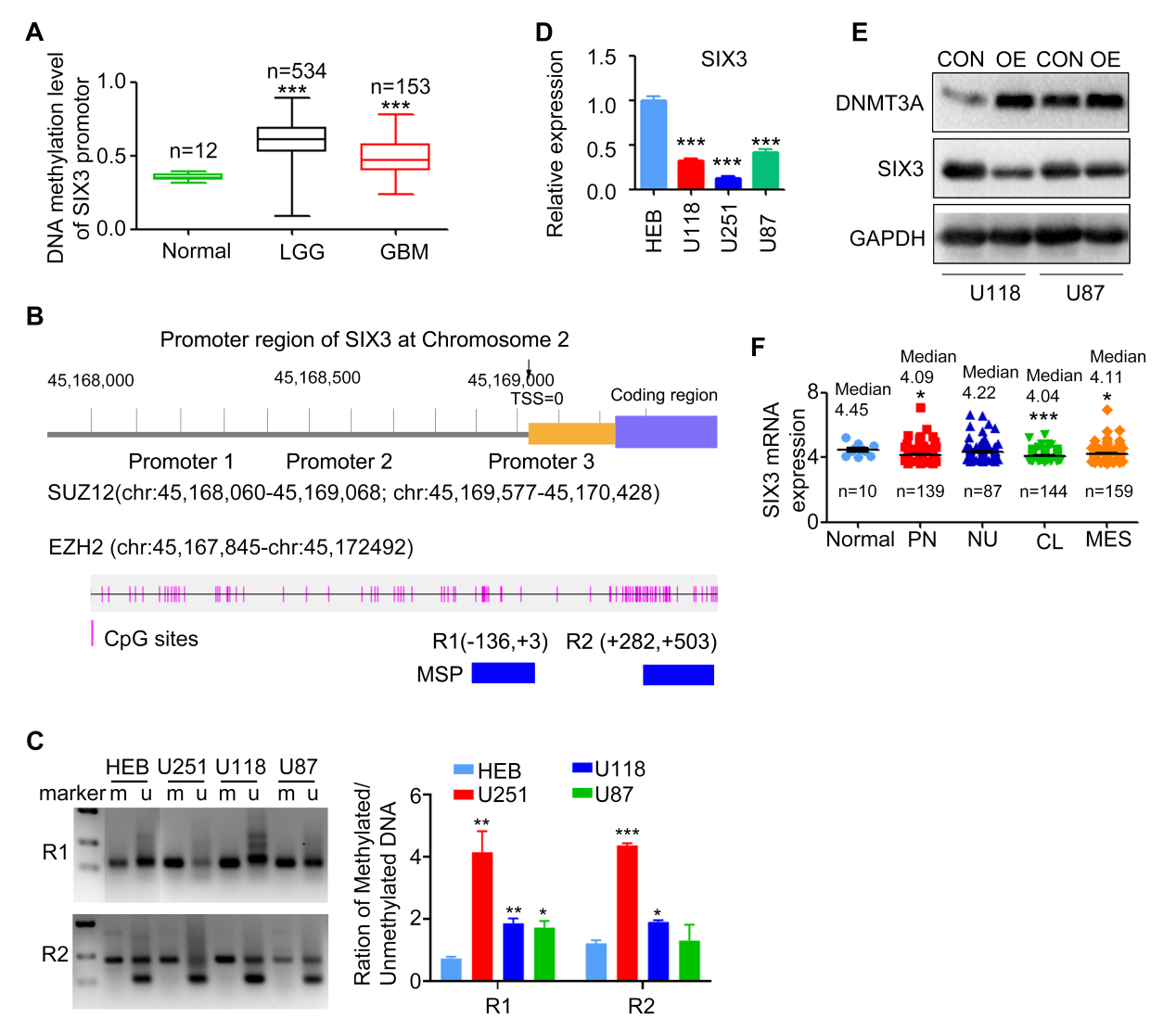
 Figure S1. SIX3 is epigenetically silenced in human glioma.**

(**A**) Statistical analysis of the DNA methylation levels of SIX3 promoter regions in glioma and normal tissues. The data were obtained from TCGA. (**B**) Schematic diagram of SIX3 promoter region. Binding regions of SUZ2，EZH2 are defined using the data from ENCODE. Promoter 1/2/3 represent the primers for ChIP-qPCR analysis. R1 and R2 represent regions for detection of methylation. (**C**) MSP detection of SIX3 promoter in HEB cells and astrocytoma cell lines. (**D**) RT-qPCR analysis of SIX3 expression in HEB cells and astrocytoma cells. (**E**) Immunoblotting analysis showing that overexpression of DNMT3A inhibited SIX3 expression in U118 and U87 cells. (**F**) Experiments form TCGA showing SIX3 expression in human glioblastoma of different molecular subtypes and in normal tissues. PN, proneural; NU, neural; CL, classical; MES, mesenchymal. (*, P<0.05; **, P<0.01; ***, P<0.001).

**
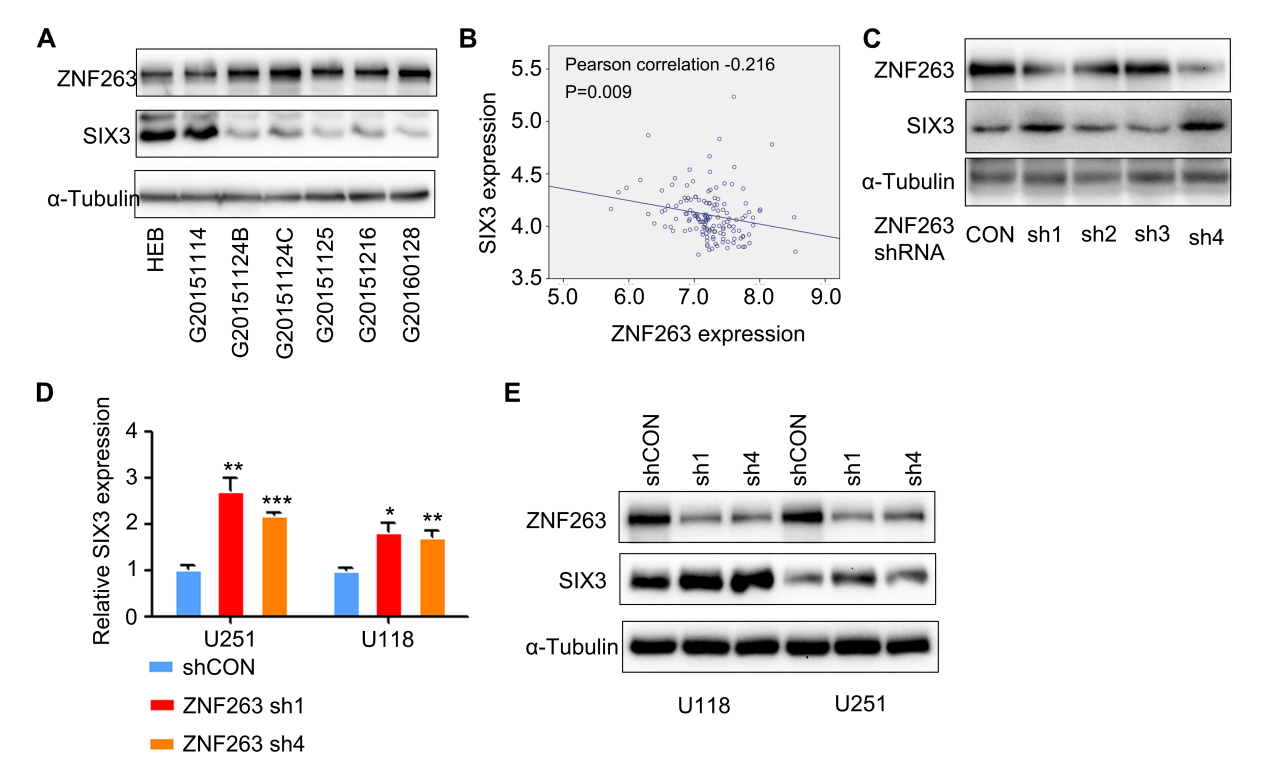
Figure S2. Inhibition of ZNF263 rescues SIX3 expression.**

1. Immunoblotting analysis showing that ZNF263 expression correlates inversely with SIX3 expression in HEB and primary human astrocytoma cells. (**B**) Correlation analysis of ZNF263 and SIX3 in Classical subtype of GBM (**C**) Immunoblotting to validate efficacy of the shRNAs targeting ZNF263 in U251 cells (**D**) RT-qPCR and (**E**) immunoblotting showing that knockdown of ZNF263 rescues SIX3 expression in human astrocytoma cells. (*, P<0.05; **, P<0.01; ***, P<0.001)


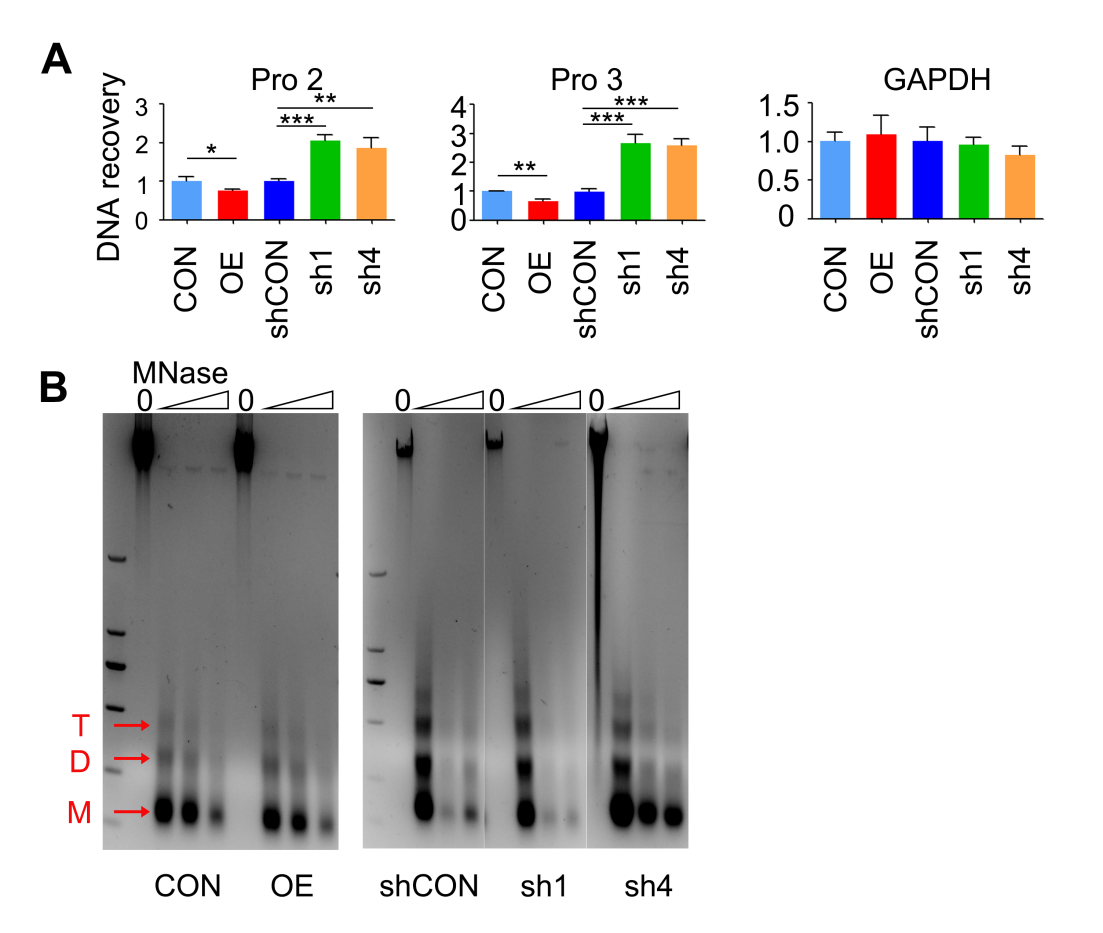


**Figure S3. ZNF263 recruits chromatin modifiers to SIX3 promoter**

(**A**) FAIRE analysis showing changes of the chromatin extractability of SIX3 promoter upon overexpression or knockdown of ZNF263. (**B**) MNase digestion assay to detect global chromatin compaction upon overexpression or knockdown of ZNF263. T: tri-nucleosome; D: di-nucleosome; M: mono-nucleosome. (*, p<0.05; **, p <0.01; ***, p <0.001)


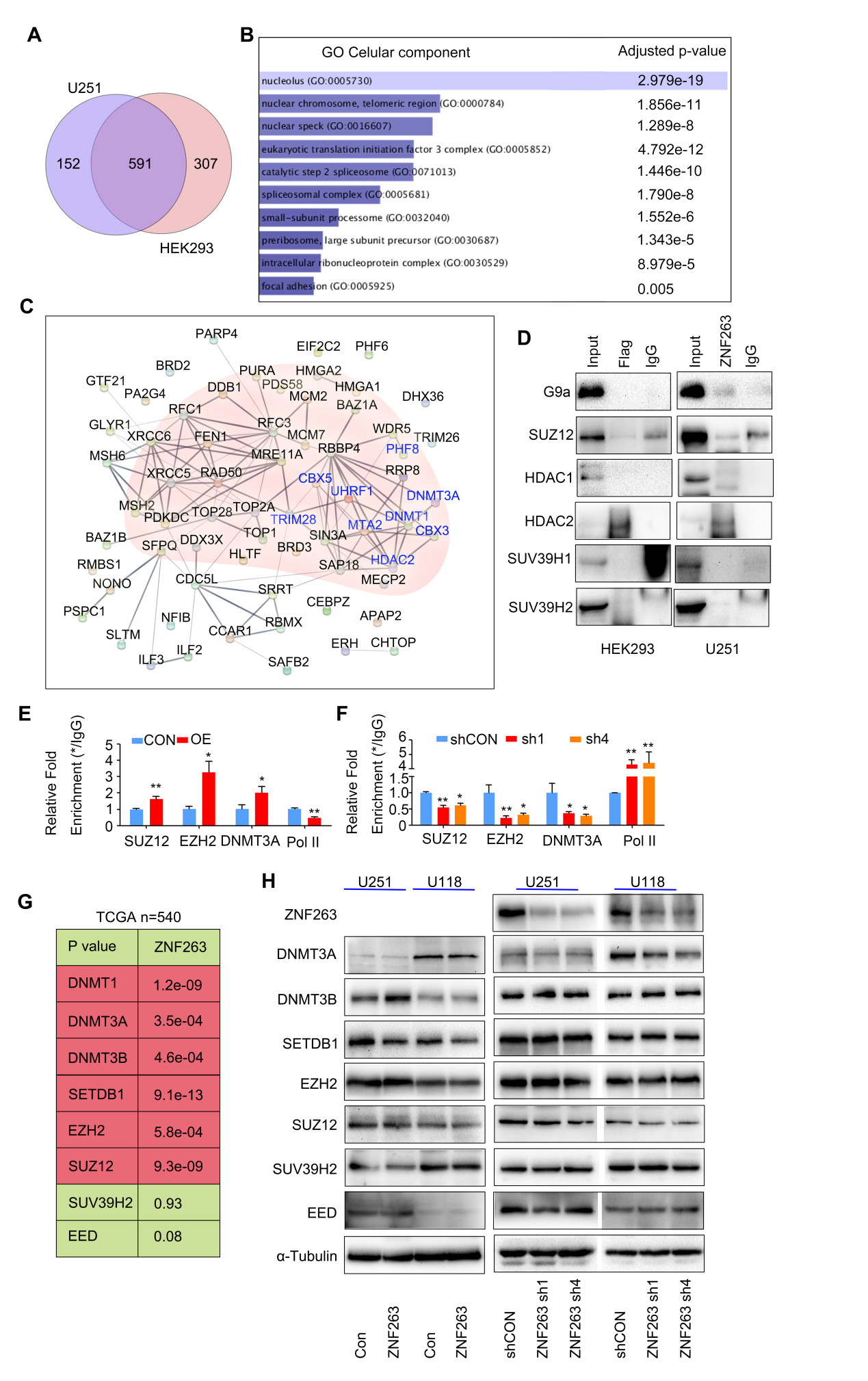


**Figure S4 ZNF263 interacts with chromatin modifiers**

(**A**) Venn diagram showing that proteins identified in U251 and HEK293 cells have high degree of overlap. Immunoprecipitation followed by tandem mass spectrometry was performed to identify proteins that interact with ZNF263. (**B**) GO analyses showing the proteins identified in ZNF263-interacting complexes are enriched in the nuclei and function in chromatin modifications, DNA transcription and RNA splicing. (**C**) String analysis showing the network of the nucleoproteins identified in ZNF263-interacting complexes. (**D**)IP followed by immunoblotting confirming proteins interacting with ZNF263. HEK293 cells were transfected with ZNF63-FLAG and DNMT3A vectors. (**E**) ChIP-qPCR analysis showing that overexpression of ZNF263 enrichment of EZH2 and DNMT3A, while reducing Polymerase II binding toSIX3 promoter. (**F**) ChIP-qPCR analysis showing that knockdown of ZNF263 increases Polymerase II binding toSIX3 promoter, but decreases recruitment of SUZ12，EZH2 and DNMT3A to SIX3 promoter (**G**) Data from with TCGA showing that ZNF263 expression correlates positively with the expression of DNMTs, SETDB1, SUZ12 and EZH2. (**H**) Immunoblotting showing that alterations in ZNF263 levels had little affects on the expression of the chromatin modifiers. (*, P<0.05; **, P<0.01; ***, P<0.001)


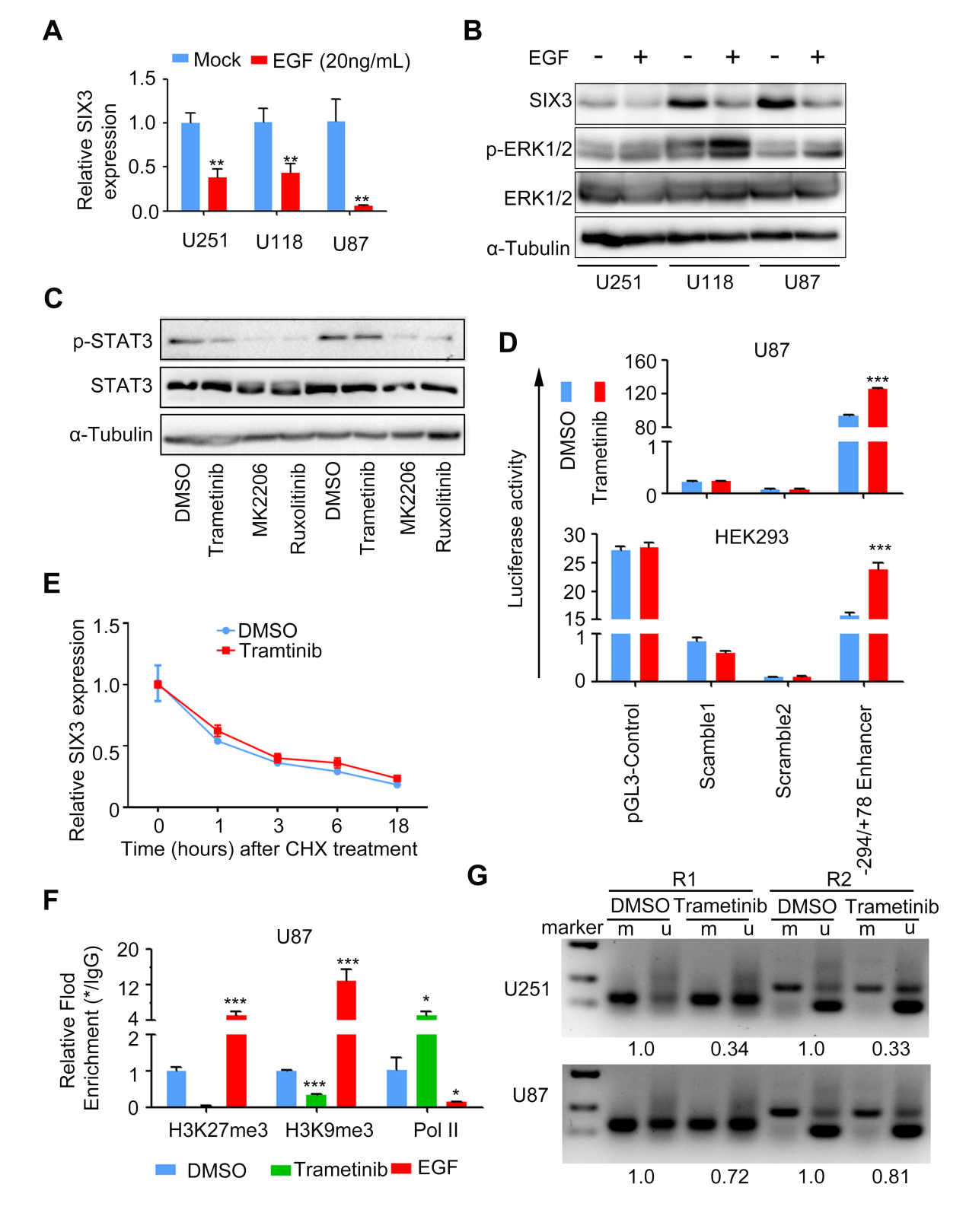


**Figure S5. Inhibition of EGFR/MAPK/ERK pathway elevates SIX3 expression and decreases chromatin modifications of SIX3 promoter.**

(**A**) RT-qPCR and (**B**) Immunoblotting showing that addition of EGF markedly decreases SIX3 expression in astrocytoma cells. (**C**) Immunoblotting showing expression of p-Stat3 and Stat3 in U251 cells treated with Trametinib (a MEK inhibitor), MK2206 (an AKT inhibitor) and Ruxolitinib (a JAK inhibitor). (**D**) Luciferase reporter assay showing that trametinib treatment specifically increases the transcriptional activity of SIX3 promoter. Scramble 1 and 2 sequences showed little promoter activity. (**E**) RT-qPCR showing the half-life of SIX3 mRNA with trametinib treatment. (**F**) ChIP-qPCR showing that trametinib treatment substantially decreases H3K7me3 and H3K9me3 in SIX3 promoter while increasing the binding of polymerase II. EGF treatment causes the opposite effect. (**G**) MSP analysis showing that trametinib treatment markedly decreases DNA methylation of SIX3 promoter. The bottom numbers represented the ration of Methylated/Unmethylated DNA. (*, P<0.05; **, P<0.01; ***, P<0.001)


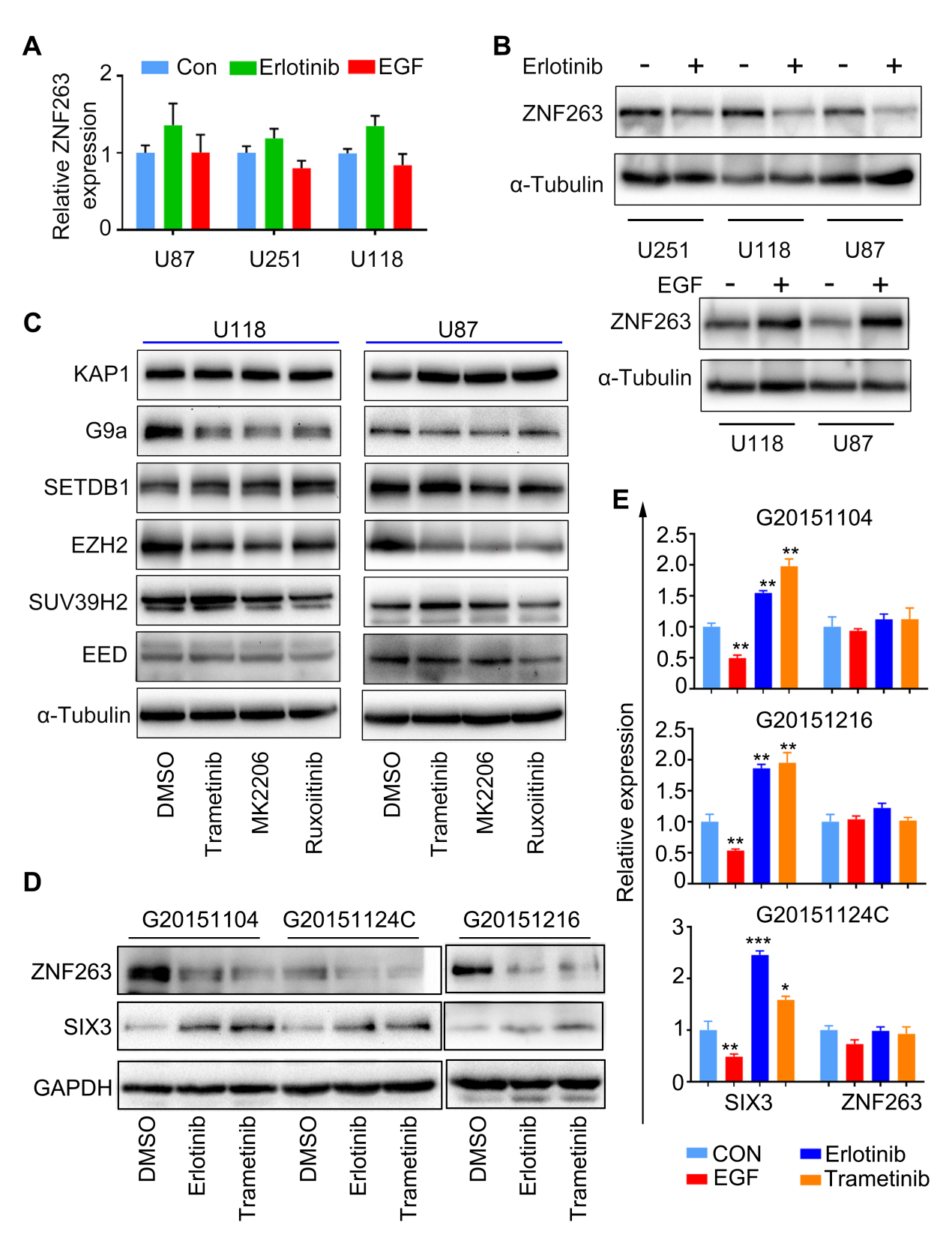


**Figure S6. Inhibition of EGFR/MAPK/ERK pathway reduces ZNF263 protein levels**

1. RT-qPCR showing ZNF263 expression in cells treated with erlotinib or EGF. (**B**) Immunoblotting showing that erlotinib treatment reduces ZNF263 protein levels (top), while EGF treatment elevates ZNF263 protein levels (bottom). (**C**) Immunoblotting showing trametinib treatment does not change the levels of KAP1, G9a and other proteins. (D) Immunoblotting showing that erlotinib or Trametinib treatment reduces ZNF263 and increases SIX3 protein levels in primary astrocytoma cells. (E) RT-qPCR showing ZNF263 and SIX3 expression in primary astrocytoma cells with erlotinib, Trametinib or EGF treatment. (*, P<0.05; **, P<0.01; ***, P<0.001)


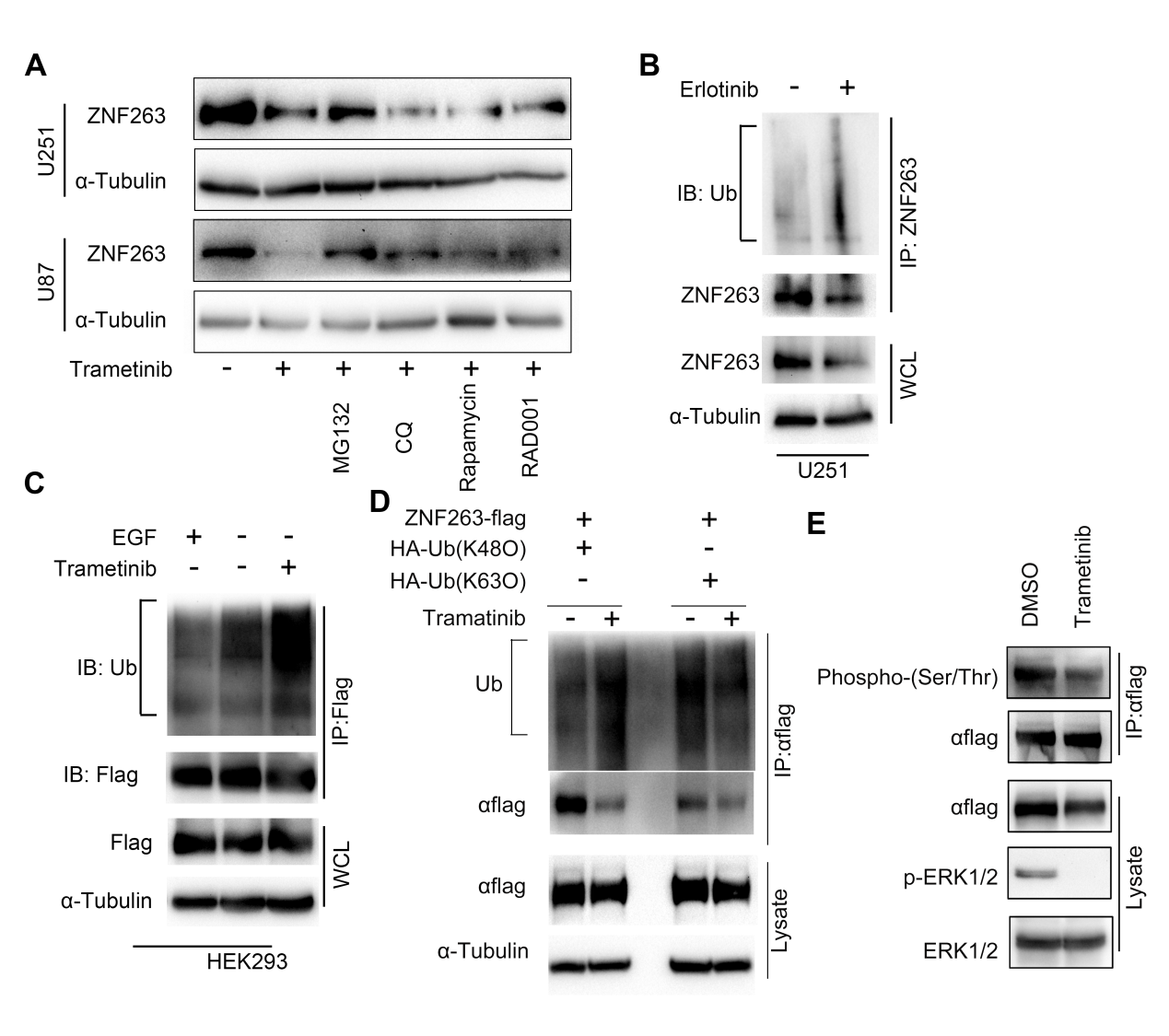


**Figure S7. ZNF263 protein is degraded through the ubiquitin proteasome pathway.**

1. Immunoblotting showing that MG132 significantly rescues the reduction of ZNF263 protein levels induced by Trametinib treatment, while inhibition of autophagy fails to do so. (**B**) Experiments with IP followed by immunoblotting showing that treatment of U251 cells with trametinib increases the ubiquitination of endogenous ZNF263. (**C**) Experiments with IP followed by immunoblotting showing that trametinib treatment increases the level of ubiquitinated ZNF263, while EGF treatment causes the opposed effect. HEK293 cells were transfected with HA-Ub and ZN263-FLAG expression vectors. (**D**) Experiments with IP and followed by immunoblotting showing that ubiquitination of ZNF263 is mediated by ubiquitination at K63. HEK293 cells were transfected with vectors containing HA-tagged Ubiquitin mutants HA-Ub-K48O and HA-Ub-K63O, together with the ZN263-FLAG expression vector. (**E**) Experiments with IP and followed by immunoblotting showing that Trametinib decreased phosphorylated ZNF263 level. (*, P<0.05; **, P<0.01; ***, P<0.001)


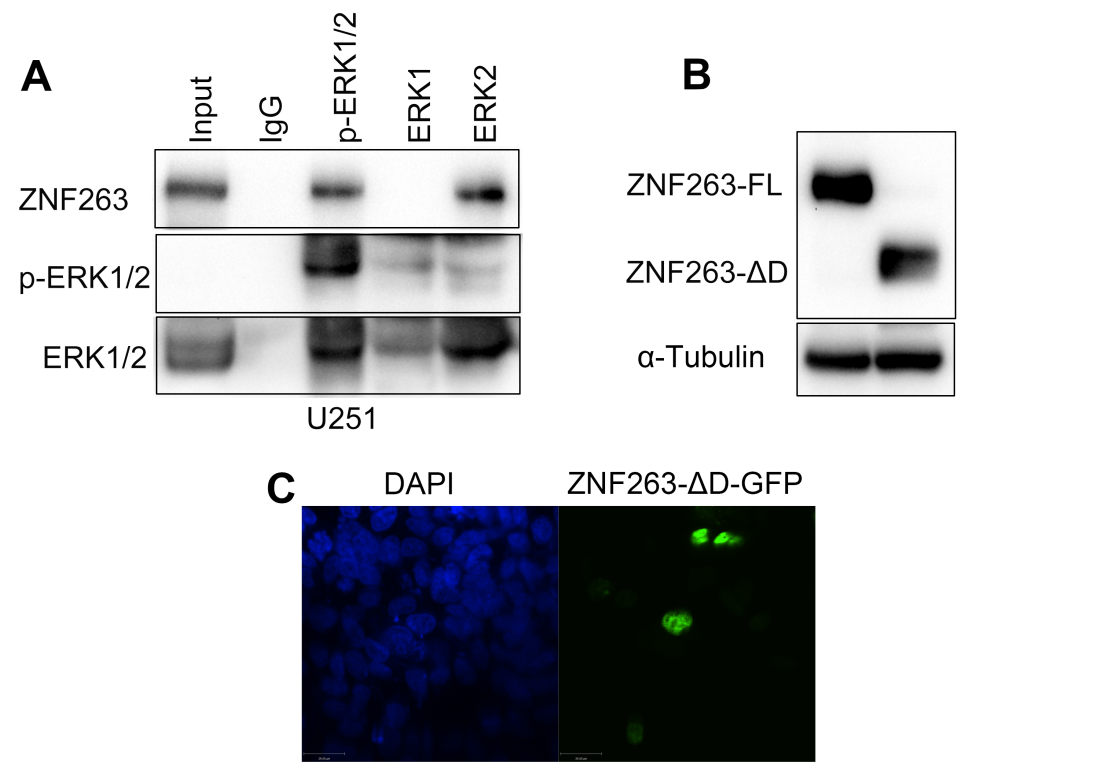


**Figure S8. ERK2 binds to ZNF263 in the nuclei.**

1. Experiments with IP followed by immunoblotting showing that ZNF263 can be detected in p-ERK1/2 and ERK2 immunoprecipitation but not in ERK1 immunoprecipitation. (**B**) Immunoblotting showing expression of ZNF263, and ZNF263-ΔD. ZNF263 and ZNF263-ΔD proteins were tagged with FLAG and detected with a FLAG antibody. (**C**) Nuclear localization of GFP-tagged ZNF263-ΔD.


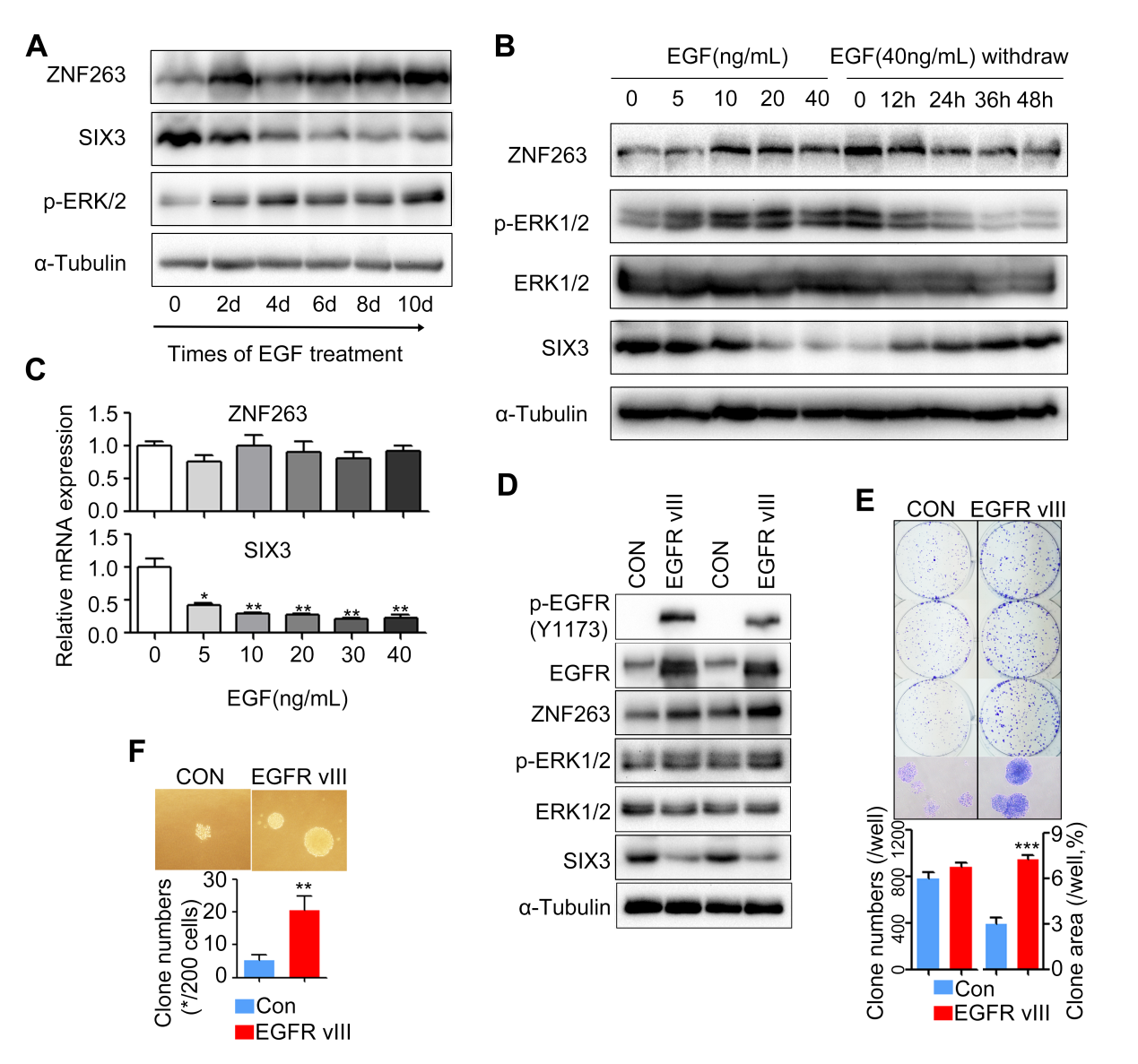


**Figure S9. The effect of EGFRvIII in HEB and astrocytoma cells**

(**A**) Immunoblotting showing that EGF treatment markedly reduces SIX3 expression and promoted ZNF263 expression. HEB cells were treated with 50ng/ml EGF for 10 days. (**B**) Immunoblotting showing that EGF treatment enhances the protein expression of ZNF263, reducing SIX3 expression. Withdrawal of EGF leads to restoration of SIX3 and ZNF263 levels. For EGF withdrawal experiment, cells were treated with 40ng/ml EGF for 60 hours, then washed with DPBS and cultured without EGF for indicated times. (**C**) RT-qPCR analysis showing that EGF treatment decreases SIX3 expression in a dose-dependent manner. In contrast, EGF has no effect on ZNF263 mRNA expression. (**D**)Immunoblotting showing expression of EGFRvIII up-regulates ZNF263, while down-regulating SIX3 in HEB cells. (**E**) and (**F**) Soft agar colony formation assay showing that EGFRvIII promotes the anchorage-independent growth of HEB cells. (*, P<0.05; **, P<0.01; ***, P<0.001)


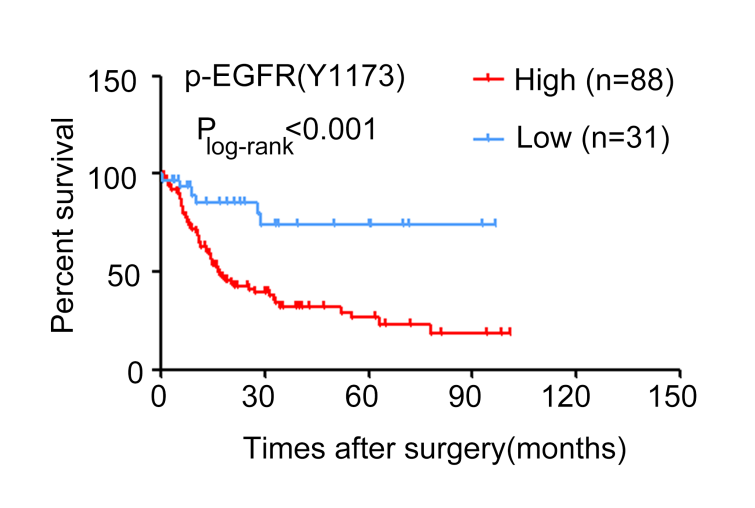


**Figure S10. Kaplan-Meier survival curves comparing survival probability among p-EGFR high expression and low expression glioma patients.**


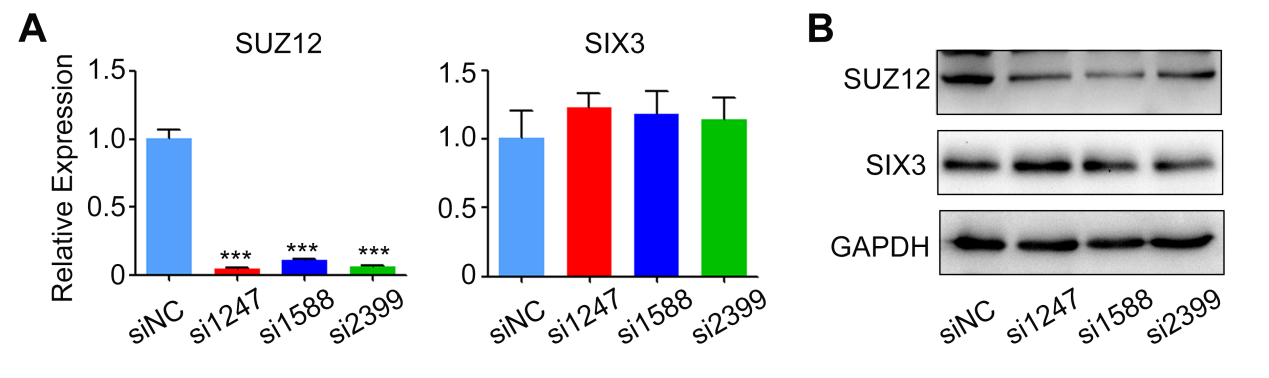


**Figure S11 SUZ12 knockdown have no effect on SIX3 expression**

**(A)** RT-qPCR showing expression of SUZ12 and SIX3 in cells transfected with control and SUZ12 siRNAs. **(B)** Immunoblotting showing SUZ12 knockdown has no effect on SIX3 protein level. (*, P<0.05; **, P<0.01; ***, P<0.001)


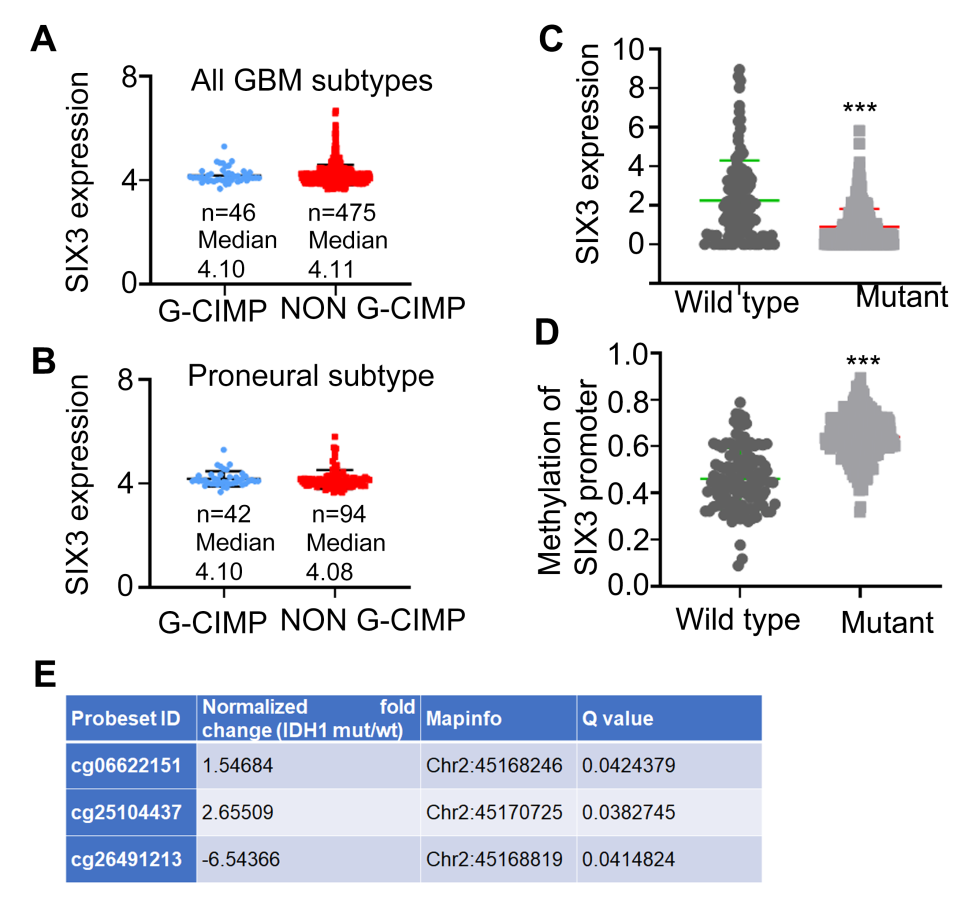


**Figure S12 Association of IDH1 mutant and SIX3 status**

(A)-(B) SIX3 expression in G-CIMP samples and NON G-CIMP samples of Total GBM (**A**) or of Proneural subtype GBM(**B**). (C) SIX3 expression in IDH1 wild type or IDH1 Mutant LGG tumors, data from TCGA. (D) SIX3 promoter methylation level in IDH1 wild type or IDH1 Mutant in LGG tumors, data from TCGA. (E) Differentially methylated CpG sites of SIX3 in mutant IDH-expressing human astrocytes. Data from Ref 1.

(*, P<0.05; **, P<0.01; ***, P<0.001)

**References:**

1. Turcan S, Rohle D, Goenka A, Walsh LA, Fang F, Yilmaz E et al. IDH1 mutation is sufficient to establish the glioma hypermethylator phenotype. Nature. 2012 Feb 15;483(7390):479-83.
