# Supplementary material for: The EGFR-ZNF263 signaling axis silences SIX3 in glioblastoma epigenetically
Source: Oncogene. 2020 Feb 13;39(15):3163–78. doi: 10.1038/s41388-020-1206-7 (PMC7142014; doi:10.1038/s41388-020-1206-7)
Supplement: Supplementary file 3 — supplementary information [file 41388_2020_1206_MOESM3_ESM.doc]

**Dear** Dr. Stebbing and Miller**:**

It is our pleasure to submit to you a manuscript entitled “The EGFR-ZNF263 Signaling Axis Silences SIX3 in Glioblastoma Epigenetically (ONC-2019-01819)” for a possible publication in your Journal.

There are no financial or other relationships that might lead to a conflict of interest.

The correspondence author for communicating with the other authors about revisions and final approval of the proofs is Professor. Minghua Wu，Cancer Research Institute, School of Basic Medical Science, Central south university, Changsha, Hunan, 410078, P.R.China. Telephone: 86-731-82355401. Fax: 86-731-82355401. E-mail: wuminghua554@aliyun.com Shuai Chen, Hunan Provincial Tumor Hospital and the Affiliated Tumor Hospital of Xiangya Medical School, Central South University ,Changsha 410013, Hunan, China Tel: 86-731-82355401. Fax: 86-731-82355401. E-mail: chenshuai16@139.com

If you need more information about our work, please let us know.

Thank you very much for your kind consideration.

Sincerely yours,

Zhibin Yu, PhD

Jianbo Feng, PhD

Professor Minghua Wu

1/29/2020
